# Significance of magnetic Reynolds number in a three-dimensional squeezing Darcy–Forchheimer hydromagnetic nanofluid thin-film flow between two rotating disks

**DOI:** 10.1038/s41598-020-74142-5

**Published:** 2020-10-14

**Authors:** Saima Riasat, Muhammad Ramzan, Seifedine Kadry, Yu-Ming Chu

**Affiliations:** 1 Department of Computer Science, Bahria University, Islamabad, 44000 Pakistan; 2 Department of Mechanical Engineering, Sejong University, Seoul, 143-747 Korea; 3 Department of Mathematics and Computer Science, Faculty of Science, Beirut Arab University, Beirut, 115020 Lebanon; 4 Department of Mathematics, Huzhou University, Huzhou, 313000 People’s Republic of China; 5 Hunan Provincial Key Laboratory of Mathematical Modeling and Analysis in Engineering, Changsha University of Science and Technology, Changsha, 410114 People’s Republic of China

**Keywords:** Software, Mechanical engineering

## Abstract

The remarkable aspects of carbon nanotubes like featherweight, durability, exceptional electrical and thermal conduction capabilities, and physicochemical stability make them desirous materials for electrochemical devices. Having such astonishing characteristics of nanotubes in mind our aspiration is to examine the squeezing three dimensional Darcy–Forchheimer hydromagnetic nanofluid thin-film flow amid two rotating disks with suspended multiwalled carbon nanotubes (MWCNTs) submerged into the base fluid water. The analysis is done by invoking partial slip effect at the boundary in attendance of autocatalytic reactions. The mathematical model consists of axial and azimuthal momentum and magnetic fields respectively. The tangential and axial velocity profiles and components of the magnetic field are examined numerically by employing the bvp4c method for varying magnetic, rotational, and squeezing Reynolds number. The torque effect near the upper and lower disks are studied critically using their graphical depiction. The values of the torque at the upper and lower disks are obtained for rotational and squeezed Reynolds numbers and are found in an excellent concurrence when compared with the existing literature. Numerically it is computed that the torque at the lower disk is higher in comparison to the upper disk for mounting estimates of the squeezed Reynolds number and the dimensionless parameter for magnetic force in an axial direction. From the graphical illustrations, it is learned that thermal profile declines for increasing values of the squeezed Reynolds number.

## Introduction

The dynamics of the magnetic field on the lubricating film have attracted the attention of researchers and scientists in recent years. It is pertinent to mention that squeezed film flow between two rotating disks with increased squeezed film pressure, load-carrying capacity, and response time as in case of classical lubrication is much fascinating area of research because of its engineering and industrial applications including jet motors, food processing, electric power generating system, turbine system, lubrication of braking devices, seismic magneto-rheological shock dampers, slider bearings, biomedical systems, and rotating machinery, etc. Squeeze film is the special regime in magnetohydrodynamics (MHD) tribology. Hughes and Elco^1^ accomplished one of the earliest analysis of the motion of incompressible viscous fluid amid two rotating disks by considering the two configurations of the magnetic field (axial and radial magnetic fields) and found that MHD interaction influences the load-carrying capability of the bearing. It is further learned that frictional torque diminishes by the supply of electricity to the electrodes in the presence of electrolytic fluid. The externally pressurized thrust bearing squeezed film case by considering the inertial effects is performed by Maki and Kumza^2^. Chawla^3^ analyzed the MHD inclined slider bearing problem with the magnetic field in the normal direction. It is detected in this study that growing Hartmann number gives rise to the substantial increase in load carrying capacities in open circuit conditions. Parakash^4^ performed the theoretical analysis of slider bearing consisting of a combination of materials for the case with applied magnetic field in the perpendicular direction to the bearing surfaces. Kamiyama^5^ considered the MHD hydrostatic thrust bearing problem with inertial effects amid two rotating disks. Agrawal^6^ presented the theoretical investigation of the inclined slider bearing problem by considering inertial effects with a transverse magnetic field. Anwar and Rodkiewicz^7^ investigated the improvement in load carrying capacity by the electric power supply to the bearing with non-uniforms magnetic field effects. This investigation reveals that this proposed experimental set up gives more improvement in load-carrying capacity even at a small Hartmann number. Soundalgekar and Amrute^8^ studied the MHD squeezed film flow between two conducting plates with a magnetic field applied in free space. They developed the relationship between the approach time and the Hartmann number. Gupta and Bhat^9^ obtained the numerical solution of the MHD porous inclined slider bearing problem with the magnetic field in the transverse direction. Patel^10^ concluded in a numerical investigation that pressure distribution, load-carrying capacities, and film thickness are the function of time and increasing slip parameter causes a decrease in load-carrying capacities. Malik and Singh^11^ considered the MHD bearing problem with the electric current applied in the axial direction and magnetic field in the perpendicular direction. The formulated problem developed the Reynolds equation which is solved by using double series expansion. The magnetic fluid between two surfaces with a magnetic field applied in the direction that is leaning to the lower surface is studied by Verma^12^. It is noticed that in the presence of magnetic fluid based squeezed film flow pressure is seen to be significantly increased. Hamza^13^ studied the effect of electromagnetic forces on the load-carrying capacity for parallel disks in the presence of a magnetic field applied in perpendicular direction. Terekhov^14^ discussed the characteristics of MHD bearing in the form of numerical series for negligible small values of magnetic Prandtl number. Lin^15^ considered the squeezed film behavior between two parallel annular disks lubricated with electrically conducting fluid flow and the magnetic field is in the transverse direction. Upon solving the Reynolds equation, it is revealed that load-carrying capacities, squeezed film pressure, and response time give a better response in the presence of a magnetic field as compared to the classical Newtonian non-conducting lubricant case. Lin^16^ further discussed the parallel rectangular plates for squeezed film characteristics. Lin et al.^17^ examined the squeezed film characteristics for annular curved disks. For the smaller values of the inner to outer radius ratio and large values of curved shaped parameter results in the enhancement in load-carrying capacity. Lu et al.^18^ used the momentum approximation method to study the MHD squeezed film characteristics by incorporating local and convective inertia. The study focused on the inertia correction factor. Bujurke and Kudenatti^19^ analyzed the squeeze film behavior by considering surface roughness. It is seen that the Reynolds equation is modified with a random rough structure. Some more studies highlighting varied features of thin-film flow may be found in^20–27^.

Nanofluids are engineered liquids comprising solid material particles submerged into the base fluid. This amalgamation has numerous applications in many industrial and engineering processes including paints, food industry, ceramics, and drug delivery procedures. The nanofluids possess ultra-cooling characteristics and are used to regulate the poor thermal performance of the customary fluids. Researchers have shown great interest in the alluring features of the nanofluids. Lu et al.^28^ studied the carbon nanotubes nanofluid flow with homogeneous- heterogeneous reactions. The numerical simulation of the flow of nanofluid amid two rotating disks with Darcy–Forchheimer effect and partial slip is studied by Hayat et al.^29^. Ramzan et al.^30^ examined the time-dependent carbon nanotubes suspended nanofluid flow amidst two extended rotating disks with combined effects of nonlinear thermal radiation and thermal stratification. Zhang et al.^31^ analyzed the effect of the magnetic Reynolds number in the presence of the gyrotactic microorganism amidst two rotating disks filled with nanofluid. Hosseinzadeh et al.^32^ also considered the motile microorganism with the cross-fluid flow over a three-dimensional cylinder. Hosseinzadeh et al.^33^ investigated the MHD hybrid nanofluid flow with different shape factors of nanoparticles. Rostami et al.^34^ performed the hydrothermal analysis of nanofluid flow with snowflake shape inner wall. The effects of MHD and nonlinear thermal radiations on a nanofluid flow with suspended carbon nanotubes with entropy generation analysis is studied by Hosseinzadeh et al.^35^. Gholinia^36^ investigated the hybrid nanofluid flow with suspended carbon nanotubes over a cylinder having a sinusoidal radius. Salehi et al.^37^ explored the MHD squeezing mixture nanofluid flow between two parallel plates. Yadav^38–40^ analyzed the MHD convection of nanofluid with suspended nanoparticles in a Hele-Shaw cell, Hall current effect in a porous media layer filled with nanofluid and the impacts of chemical reactions on convective heat transfer in nanofluid with a porous enclosure. Zuo et al.^41^ accomplished the thermal investigation of hybrid nanoparticles with different permeabilities. Lu et al.^42^ considered the nanofluid flow by considering homogeneous and heterogeneous reactions. Ahmed et al.^43^ employed the FEM-CBS algorithm for convective transport of nanofluids. Some more recent studies highlighting various aspects are appended at^44–51^.

Given the foregoing, it is revealed that very few articles are available discussing the significance of magnetic Reynolds number in numerous geometries. Nevertheless, none of these has discussed the nanofluid thin-film flow comprising multi-walled carbon nanotubes between two rotating disks with the impact of magnetic Reynolds number. The additional features that distinguish the envisioned mathematical model from existing literature are the Darcy–Forchheimer effect with autocatalytic chemical reaction and partial slip at the boundary. The flow problem is tackled with the numerical scheme bvp4c. The impacts of pertinent parameters versus the associated profiles are depicted through the graphs with logical deliberations. The present results in the limiting case are also validated through numerical calculations. An excellent correlation between the results is found.

The present study facilitates the existing literature to answer the following critical questions:

- What is the impact of magnetic Reynolds number on the axial and tangential components of the induced magnetic field?
- How rotation affects the axial velocity profile?
- What is the significance of nanofluid thin-film flow comprising multiwalled carbon nanotubes in the envisioned mathematical model?
- How thermal profile is affected by the Prandtl number?
- What is the impact of the relative rotation parameter on the amplitude of the tangential velocity profile?
- How the concentration of the nanofluid film flow is affected by the autocatalytic chemical reaction?

## Mathematical formulation

**Figure 1:**
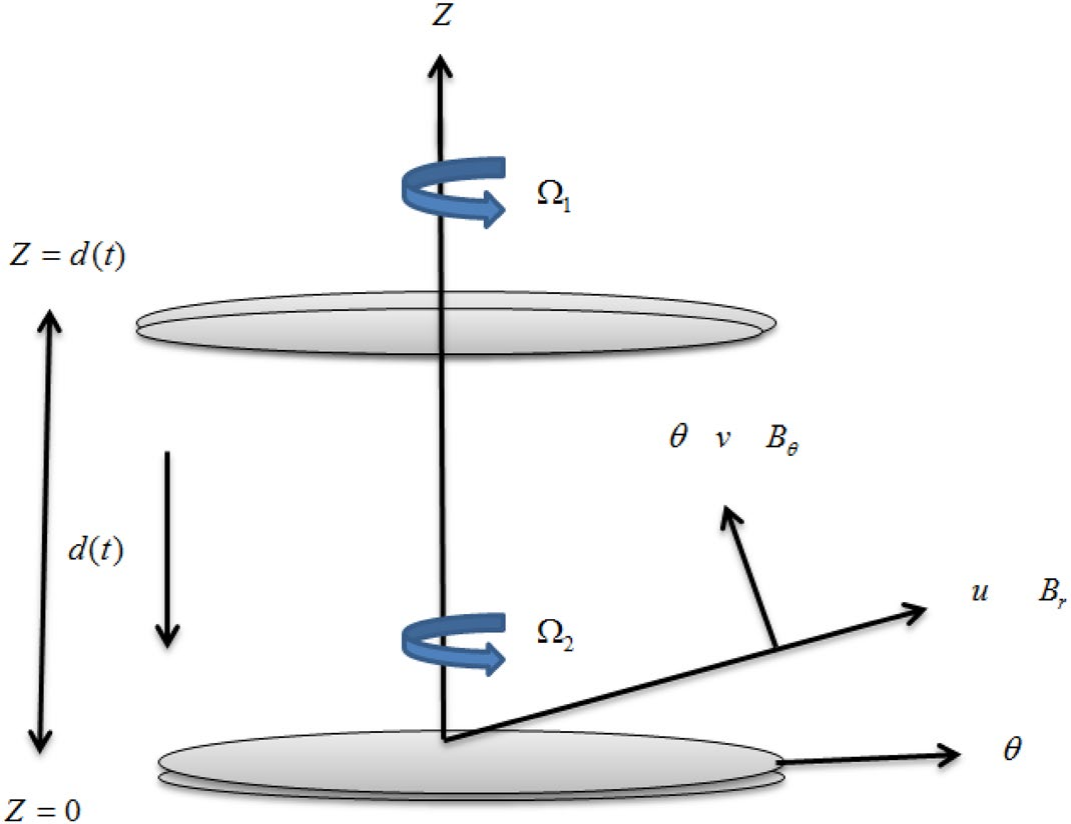
Schematic diagram for the squeeze nanofluid film flow regime.

Consider an axisymmetric squeezed nanofluid thin-film flow comprising multi-walled carbon nanotubes amidst two rotating disks. The velocity ***V*** with its components (*u*, *v*, *w*) is taken in the (*r*, *θ*, *z*) directions respectively. Both disks are separated by the distance $d(t) = D(1 - \alpha \zeta )^{1/2}$ at the time *t*, with *D* is the representative length at *t* = 0 and *α*^−1^ signifies the time. The lower disk is fixed but the upper disk is moving towards the lower. The axis of symmetry is taken as *z*-axis about which the upper and the lower disks are rotating at the rates $\frac{{\Omega_{1} }}{1 - \alpha \zeta }$ and $\frac{{\Omega_{2} }}{1 - \alpha \zeta }$ respectively, with Ω_1_ and Ω_2_ denote the angular velocities having dimension *t*^−1^. This means that the lower disk can rotate but can’t move in the axial direction, however, the upper disk can rotate and move along its axis of symmetry. The external applied magnetic field on the upper disk is represented by ***H*** and has the tangential and axial components denoted by $H_{\theta } = \frac{{rN_{o} }}{{\mu_{2} (1 - \alpha \zeta )}}$ and $H_{z} = \frac{{ - \alpha M_{o} }}{{\mu_{1} (1 - \alpha \zeta )^{1/2} }}$ respectively defined by El-Shekh et al.^22^. Here, *M*_*o*_ and *N*_*o*_ magnetic field quantities which make *H*_*θ*_ and *H*_*z*_ dimensionless and *μ*_2_, *μ*_1_ are the magnetic permeabilities of squeezed film and medium external to the disk respectively. The induced magnetic field ***B***(*r*, *θ*, *z*) with components ***B*** = (*B*_*r*_, *B*_*θ*_, *B*_*z*_) is instigated by the applied magnetic field between two rotating disks in a thin nanofluid film flow. The schematic diagram of the flow pattern is depicted in Fig. 1.

The mathematical model used for autocatalytic chemical reaction^28^ is given by:

1 $$ A^{*} + 2B^{*} \to 3B^{*} ,{\text{ rate}} = k_{c} ab^{2} , $$

2 $$ A^{*} \to B^{*} ,\quad {\text{rate}} = k_{s} a, $$

where *a* and *b* are the respective concentrations of the chemical species A* and B*.

The mass conservation equation^26^ is represented by:

3 $$ div{\varvec{V}} = 0, $$

and the equations for magnetic field are^26^:

4 $$ {\varvec{B}} = \mu_{2} \user2{H,} $$

5 $$ div{\varvec{B}} = 0, $$

6 $$ \frac{{\partial {\varvec{B}}}}{\partial t} = \nabla \times ({\varvec{V}} \times {\varvec{B}}) + \frac{1}{{\sigma_{nf} \mu_{2} }}\nabla^{2} {\varvec{B}} $$

The magnetic field influences the moving charged particles. The relation between applied and induced magnetic field is represented by Eq. (4). Moving charges experience a Lorentz force, which is given by ***F*** = *q*(***E*** + ***V*** × ***B***), where *q* represents the electric charge and ***E*** is the electric field.

Modified Naiver- Stokes equations are given by^26^:

7 $$ \frac{{\partial {\varvec{V}}}}{\partial t} + ({\varvec{V}}.\nabla ){\varvec{V}} = - \frac{1}{{\rho_{nf} }}\nabla p + \nu_{nf} \nabla^{2} {\varvec{V}} + \frac{1}{{\rho_{nf} \mu_{2} }}\nabla \times ({\varvec{V}} \times {\varvec{B}}), $$

where *p* denotes the hydromagnetic pressure. $\rho_{nf}$ and $\nu_{nf}$ denote the nanofluid density and viscosity respectively.

The energy equation is defined as^31^:

8 $$ (\rho C_{p} )_{nf} ({\varvec{V}}.\nabla )\user2{T = }\nabla .(k_{nf} \nabla ){\varvec{T}}, $$

Momentum conservation equation takes the following form

9 $$ \frac{\partial u}{{\partial t}} - \frac{{v^{2} }}{r} = - \frac{1}{{\rho_{nf} }}\frac{\partial p}{{\partial r}} + \nu_{nf} \frac{{\partial^{2} u}}{{\partial z^{2} }} + \frac{1}{{\rho_{nf} \mu_{2} }}\left(2rM\frac{{\partial^{2} M}}{{\partial z^{2} }} - rN^{2} \right) - \nu_{nf} \frac{u}{k} - \frac{{Fu^{2} }}{\sqrt k }, $$

10 $$ \frac{\partial v}{{\partial t}} + u\frac{\partial v}{{\partial r}} + w\frac{\partial v}{{\partial z}} + \frac{vu}{r} = \nu_{nf} \frac{{\partial^{2} v}}{{\partial z^{2} }} - \frac{1}{{\rho_{nf} \mu_{2} }}\left( {2rM\frac{\partial N}{{\partial z}} - 4rN\frac{\partial M}{{\partial z}}} \right) - \nu_{nf} \frac{v}{k} - \frac{{Fv^{2} }}{\sqrt k }, $$

11 $$ \nu_{nf} \frac{{\partial^{2} w}}{{\partial z^{2} }} - \frac{1}{{\rho_{nf} \mu_{2} }}\left( {r^{2} N\frac{\partial N}{{\partial z}} - r^{2} \frac{{\partial^{3} M}}{{\partial z^{3} }}} \right) - \nu_{nf} \frac{w}{k} - \frac{{Fw^{2} }}{\sqrt k } = 0, $$

The conservation equation for the induced magnetic field takes the following form^26^:

12 $$ \frac{{\partial B_{r} }}{\partial t} = - r\frac{{\partial^{3} M}}{{\partial z^{3} }} + \frac{1}{{\sigma_{nf} \mu_{2} }}\frac{{\partial^{2} B_{r} }}{{\partial z^{2} }}, $$

13 $$ \frac{{\partial B_{\theta } }}{\partial t} = - r\frac{{\partial^{2} N}}{{\partial z^{2} }} + \frac{1}{{\sigma_{nf} \mu_{2} }}\frac{{\partial^{2} B_{\theta } }}{{\partial z^{2} }} $$

14 $$ \frac{{\partial B_{z} }}{\partial t} = - \frac{1}{{\sigma_{nf} \mu_{2} }}\frac{{\partial^{2} B_{z} }}{{\partial z^{2} }} $$

The equation for the homogeneous- heterogeneous reaction is^28^:

15 $$ \frac{\partial a}{{\partial t}} + u\frac{\partial a}{{\partial r}} + w\frac{\partial a}{{\partial z}} = D_{A} \left( {\frac{{\partial^{2} a}}{{\partial r^{2} }} + \frac{1}{r}\frac{\partial a}{{\partial r}} + \frac{{\partial^{2} a}}{{\partial z^{2} }}} \right) - k_{c} ab^{2} , $$

16 $$ \frac{\partial b}{{\partial t}} + u\frac{\partial b}{{\partial r}} + w\frac{\partial b}{{\partial z}} = D_{A} \left( {\frac{{\partial^{2} b}}{{\partial r^{2} }} + \frac{1}{r}\frac{\partial b}{{\partial r}} + \frac{{\partial^{2} b}}{{\partial z^{2} }}} \right) + k_{c} ab^{2} , $$

with the boundary conditions:

17 $$ u = A^{\prime}u_{z} ,\quad v = \frac{{r\Omega_{2} }}{1 - \alpha \zeta } + B^{\prime}v_{z} ,\quad w = 0,\quad T = T_{1} ,\quad B_{\theta } = B_{z} = 0,\quad D_{A} \frac{\partial a}{{\partial z}} = k_{s} a,\quad D_{B} \frac{\partial b}{{\partial z}} = - k_{s} a,\quad at\quad z = 0, $$

18 $$ u = 0,\quad v = \frac{{r\Omega_{1} }}{1 - \alpha \zeta },\quad w = \frac{ - \alpha D}{{2\sqrt {(1 - \alpha \zeta )} }},\quad T = T_{u} ,\quad B_{\theta } = \frac{{rN_{o} }}{(1 - \alpha \zeta )} = B_{z} = \frac{{ - \alpha M_{o} }}{{\sqrt {(1 - \alpha \zeta )} }},\quad a \to a_{\infty } ,\quad b \to 0,\quad {\text{as}}\quad z \to \infty . $$

Thermo-physical traits of multiwall carbon nanotubes (MWCNTs) are^28^:

19 $$ A = \frac{{\mu_{nf} }}{{\mu_{f} }} = \frac{1}{{\left( {1 - \psi } \right)^{2.5} }} $$

20 $$ B = \frac{{\rho_{nf} }}{{\rho_{f} }} = \left( {1 - \psi } \right) + \frac{{\rho_{CNT} }}{{\rho_{f} }}\psi $$

21 $$ C = \frac{{(\rho C_{p} )_{nf} }}{{(\rho C_{p} )_{f} }} = (1 - \psi ) + \frac{{(\rho C_{p} )_{CNT} }}{{(\rho C_{p} )_{f} }}\psi $$

22 $$ D = \frac{{k_{nf} }}{{k_{f} }} = \frac{{\left( {1 - \psi } \right) + 2\psi \frac{{k_{CNT} }}{{k_{CNT} - k_{f} }}ln\frac{{k_{CNT} + k_{f} }}{{2k_{f} }}}}{{\left( {1 - \psi } \right) + 2\psi \frac{{k_{f} }}{{k_{CNT} - k_{f} }}ln\frac{{k_{CNT} + k_{f} }}{{2k_{f} }}}} $$

23 $$ A_{2} = \frac{{\sigma_{nf} }}{{\sigma_{f} }} = 1 + \frac{{3\psi \left( {\frac{{\sigma_{CNT} }}{{\sigma_{f} }} - 1} \right)}}{{\left( {\frac{{\sigma_{CNT} }}{{\sigma_{f} }} + 2} \right) - \left( {\frac{{\sigma_{CNT} }}{{\sigma_{f} }} - 1} \right)}} $$

Table 1 represents thermos-physical features of H_2_O and MWCNTs^28^.

**Table 1:** The thermophysical attributes of MWCNTs and water.

Properties	MWCNT	Water
$k\left( {\frac{W}{{{\text{mK}}}}} \right)$	3000	0.613
$\rho \left( {\frac{{{\text{kg}}}}{{{\text{m}}^{3} }}} \right)$	1600	997.1
$C_{p} \left( {\frac{{{\text{J}}\,{\text{K}}}}{{{\text{kg}}}}} \right)$	796	4179
$\sigma (\Omega \;{\text{m}})^{ - 1}$	1 × 10^7^	0.05

Introducing the following transformations Hamza^24^ and ElShekh et al.^22^:

24 $$ \eta \user2{ = }\frac{z}{d(t)}, $$

25 $$ d(t) = D(1 - \alpha \zeta )^{1/2} $$

26 $$ u = r\frac{\partial F(z,t)}{{\partial z}} = \frac{{\alpha r\frac{df}{{d\eta }}}}{2(1 - \alpha \zeta )}, $$

27 $$ v = rG(z,t) = \frac{{\Omega_{1} rg(\eta )}}{(1 - \alpha \zeta )}, $$

28 $$ w = - 2F(z,t) = \frac{\alpha Df(\eta )}{{(1 - \alpha \zeta )^{1/2} }}, $$

29 $$ B_{r} = r\frac{\partial M(z,t)}{{\partial z}} = \frac{{\alpha rM_{o} \frac{dm}{{d\eta }}}}{2D(1 - \alpha \zeta )}, $$

30 $$ B_{\theta } = rN(z,t) = \frac{{rN_{o} n(\eta )}}{2(1 - \alpha \zeta )}, $$

31 $$ B_{z} = - 2M(z,t) = - \frac{{\alpha M_{o} m(\eta )}}{{(1 - \alpha \zeta )^{1/2} }}, $$

32 $$ a = c_{0} \tilde{\varphi },b = c_{0} \tilde{l}. $$

Using Eqs. (24)–(32), we get the following system of highly coupled nonlinear ordinary differential equations with one independent variable *η*.

The transformed ordinary differential equations are:

33 $$ \begin{aligned} & \frac{{2BR_{2} }}{A}\left[ {\left( {3 + \frac{A}{2B}\lambda } \right)f^{\prime \prime } + \eta f^{\prime \prime \prime } - 2\left( {\frac{{R_{1} }}{{R_{2} }}} \right)^{2} gg^{\prime } + \frac{{2R_{3}^{2} }}{B}mm^{\prime \prime \prime } } \right. \\ & \left. {\quad + \frac{{2R_{4}^{2} }}{B}\left( {\frac{{R_{1} }}{{R_{2} }}} \right)^{2} nn^{\prime } + 2F_{r} f^{\prime } f^{\prime \prime } } \right] = f^{iv} , \\ \end{aligned} $$

34 $$ R_{2} \left[ {\left( {2 + \frac{A}{B}\lambda } \right)g + \eta g^{\prime} + 2f^{\prime}g - 2fg^{\prime} + 2R_{3} R_{4} \left( {mn^{\prime} - nm^{\prime}} \right) + F_{r} g^{2} } \right] = g^{\prime\prime}, $$

35 $$ m^{\prime \prime \prime } = {\text{Re}}_{m} [2m^{\prime} + \eta m^{\prime\prime} - 2fm^{\prime\prime} + 2mf^{\prime\prime}], $$

36 $$ n^{\prime\prime} = {\text{Re}}_{m} \left[ {2n + \eta n^{\prime} - 2fn^{\prime} + 2\left( {\frac{{R_{3} }}{{R_{4} }}} \right)mg^{\prime}} \right], $$

37 $$ \frac{C}{D}R_{2} \Pr (\eta \theta ^{\prime} - 2f\theta ^{\prime}) = \theta ^{\prime\prime}, $$

38 $$ \frac{1}{Sc}h^{\prime\prime} - \frac{\eta }{2}h^{\prime} + fh^{\prime} - k_{1} h(1 - h)^{2} = 0 $$

The dimensionless parameters appearing in the resulting ordinary differential equations are translated as:

39 $$ \begin{aligned} & \lambda = \frac{{\nu_{f} (1 - \alpha \zeta )}}{\alpha k},\quad Fr = \frac{{C_{b}^{*} }}{{k^{1/2} }}r,\quad k_{1} = \frac{{k_{c} a_{o}^{2} (1 - \alpha \zeta )}}{\alpha },\quad k_{2} = \frac{{\alpha D^{2} }}{{D_{A} }},\quad R_{1} = \frac{{\Omega_{1} D^{2} }}{{\nu_{f} }}, \\ & R_{2} = \frac{{\alpha D^{2} }}{{2\nu_{f} }},\quad R_{3} = \frac{{M_{o} }}{{D\sqrt {\mu_{2} \rho_{f} } }},\quad R_{4} = \frac{{N_{o} }}{{\Omega_{1} \sqrt {\mu_{2} \rho_{f} } }},\quad {\text{Re}}_{m} = R_{2} Bt,\quad Bt = \sigma_{f} \mu_{2} \nu_{f} , \\ & \Pr = \frac{{(\rho C_{p} )_{f} v_{f} }}{{k_{f} }},\quad Sc = \frac{{\alpha D^{2} }}{{D_{A} }},\quad S = \frac{{\Omega_{2} }}{{\Omega_{1} }} \\ \end{aligned} $$

with the following boundary conditions

40 $$ \begin{aligned} f^{\prime}(0) & = A_{1} f^{\prime\prime}(0),\quad g(0) = 1 + C_{1} g^{\prime}(0),\quad f(0) = 0,\quad \theta (0) = 1,\quad m(0) = 0,\quad n(0) = 0,\quad h^{\prime}(0) = k_{2} h(0), \\ f^{\prime}(\infty ) & = 0,\quad g(\infty ) = \frac{{\Omega_{2} }}{{\Omega_{1} }} = S,\quad f(\infty ) = \frac{1}{2},\quad \theta (\infty ) = 0,\quad m(\infty ) = 1,\quad n(\infty ) = 1,\quad h(1) \to 1, \\ \end{aligned} $$

where *Fr* stands for Forchheimer number, *k*_1_, *k*_2_ are the measure of the strength of homogeneous and heterogeneous reaction respectively. *R*_1_ is the rotational Reynolds number, *R*_2_ is the squeeze Reynolds number. The numbers *R*_3_ and *R*_4_ are the dimensionless parameters for magnetic force in axial and tangential direction respectively. Re_*m*_ is the magnetic Reynolds number. *Bt* is the Batcheler number. It is pertinent to mention that *t*^−1^ = *α*, is the inverse time^26^.

From the tribological application point of view, we have emphasized on calculating the torque at the upper disk as:

41 $$ \tau_{u} = 2\pi \mu_{f} \int\limits_{0}^{a} {\left[ {\frac{\partial v}{{\partial z}}} \right]}_{z = d} dr, $$

The dimensionless torque at the upper disk is the tangential velocity gradient and is given by:

42 $$ \tilde{\tau }_{u} = \frac{{2D(1 - \alpha \zeta )^{3/2} }}{{\pi \mu_{f} \Omega_{1} a^{4} }}\tau_{u} = \frac{dg(1)}{{d\eta }}, $$

Similarly, we can calculate torque at lower disk given by:

43 $$ \tilde{\tau }_{lower} = \frac{dg(0)}{{d\eta }}, $$

## Numerical procedure

The system of Eqs. (33)–(38) assisted by the boundary conditions (40) is translated to the 1^st^ order differential equations’ system and solved by employing MATLAB software function bvp4c. A tolerance of 10^–6^ is fixed for the initial approximations to obtain a numerical solution. This assumed preliminary guess must satisfy Eq. (40) without disturbing the solution. The bvp4c method is implemented to evaluate the transformed coupled non-linear ordinary differential equations. First, new variables are introduced to obtain the system of first-order equations:

44 $$ \begin{aligned} & f = Y_{1} ,\quad f^{\prime} = Y_{2} ,\quad f^{\prime\prime} = Y_{3} ,\quad f^{\prime \prime \prime } = Y_{4} ,\quad f^{iv} = yy_{1} ,\quad g = Y_{5} ,\quad g^{\prime} = Y_{6} ,\quad g^{\prime\prime} = Y_{7} , \\ & g^{\prime \prime \prime } = yy_{2} ,\quad m = Y_{8} ,\quad m^{\prime} = Y_{9} ,\quad m^{\prime\prime} = yy_{3} ,\quad n = Y_{10} ,\quad n^{\prime} = Y_{11} ,\quad n^{\prime\prime} = yy_{4} ,\quad \theta = Y_{12} ,\quad \theta ^{\prime} = Y_{13} ,\quad \theta ^{\prime\prime} = yy_{5} , \\ & h = Y_{14} ,\quad h^{\prime} = Y_{15} ,\quad h^{\prime\prime} = yy_{6} . \\ \end{aligned} $$

Using the above equations, the transformed first-order differential equation with boundary conditions are:

45 $$ \begin{aligned} yy_{1} & = \frac{{2BR_{2} }}{A}\left[ {\left( {3 + \frac{A}{2B}\lambda } \right)Y_{3} + \eta Y_{4} - 2\left( {\frac{{R_{1} }}{{R_{2} }}} \right)^{2} Y_{5} Y_{6} + \frac{{2R_{3}^{2} }}{B}_{7} \left( {{\text{Re}}_{m} \left( {2Y_{10} + \eta Y_{11} - 2Y_{1} Y_{11} + Y_{7} Y_{11} \frac{{2R_{3} }}{{R_{4} }}} \right)} \right.} \right. \\ & \left. {\quad + \frac{{2R_{4}^{2} }}{B}\left( {\frac{{R_{1} }}{{R_{2} }}} \right)^{2} Y_{10} Y_{11} + 2F_{r} Y_{3} Y_{2} } \right], \\ \end{aligned} $$

46 $$ yy_{2} = R_{2} \left[ {\left( {2 + \frac{A}{B}\lambda } \right)Y_{5} + \eta Y_{6} + 2Y_{2} Y_{5} - 2Y_{1} Y_{6} + 2R_{3} R_{4} \left( {Y_{7} Y_{11} - Y_{10} Y_{8} } \right) + FrY_{5}^{2} } \right] $$

47 $$ yy_{3} = {\text{Re}}_{m} [2Y_{8} + \eta \times Y_{9} - 2Y_{1} Y_{9} + 2Y_{7} Y_{3} ], $$

48 $$ yy_{4} = {\text{Re}}_{m} \left[ {2Y_{10} + Y_{11} - 2Y_{1} Y_{11} + Y_{7} Y_{11} \left( {\frac{{R_{3} }}{{R_{4} }}} \right)} \right], $$

49 $$ yy_{5} = \frac{{CR_{2} \Pr }}{D}\left[ {\eta \times Y_{13} - 2Y_{1} Y_{13} } \right], $$

50 $$ yy_{6} = Sc\left[ {\frac{\eta }{2} \times Y_{15} - Y_{1} Y_{15} - k_{1} Y_{14} (1 - Y_{14} )^{2} } \right], $$

51 $$ \begin{aligned} & Y_{1} (0) = 0,\quad Y_{2} (0) = A,\quad Y_{3} (0) = 0,\quad Y_{5} (0) = 1 + c,\quad Y_{6} (0) = 0,\quad Y_{7} (0) = 0,\lambda ,\quad Y_{10} (0) = 0, \\ & Y_{12} (0) = 1,\quad Y_{14} (0) = k_{2} ,\quad Y_{15} (0) = k_{2} Y_{14} (0),\quad Y_{1} (\infty ) = 0.5,\quad Y_{2} (\infty ) = 0,\quad Y_{5} (\infty ) - S,\quad Y_{7} (\infty ) = 1 \\ & Y_{10} (\infty ) = 1,\quad Y_{12} (\infty ) = 0,\quad Y_{14} (\infty ) = 1. \\ \end{aligned} $$

## Results and discussion

**Figure 2:**
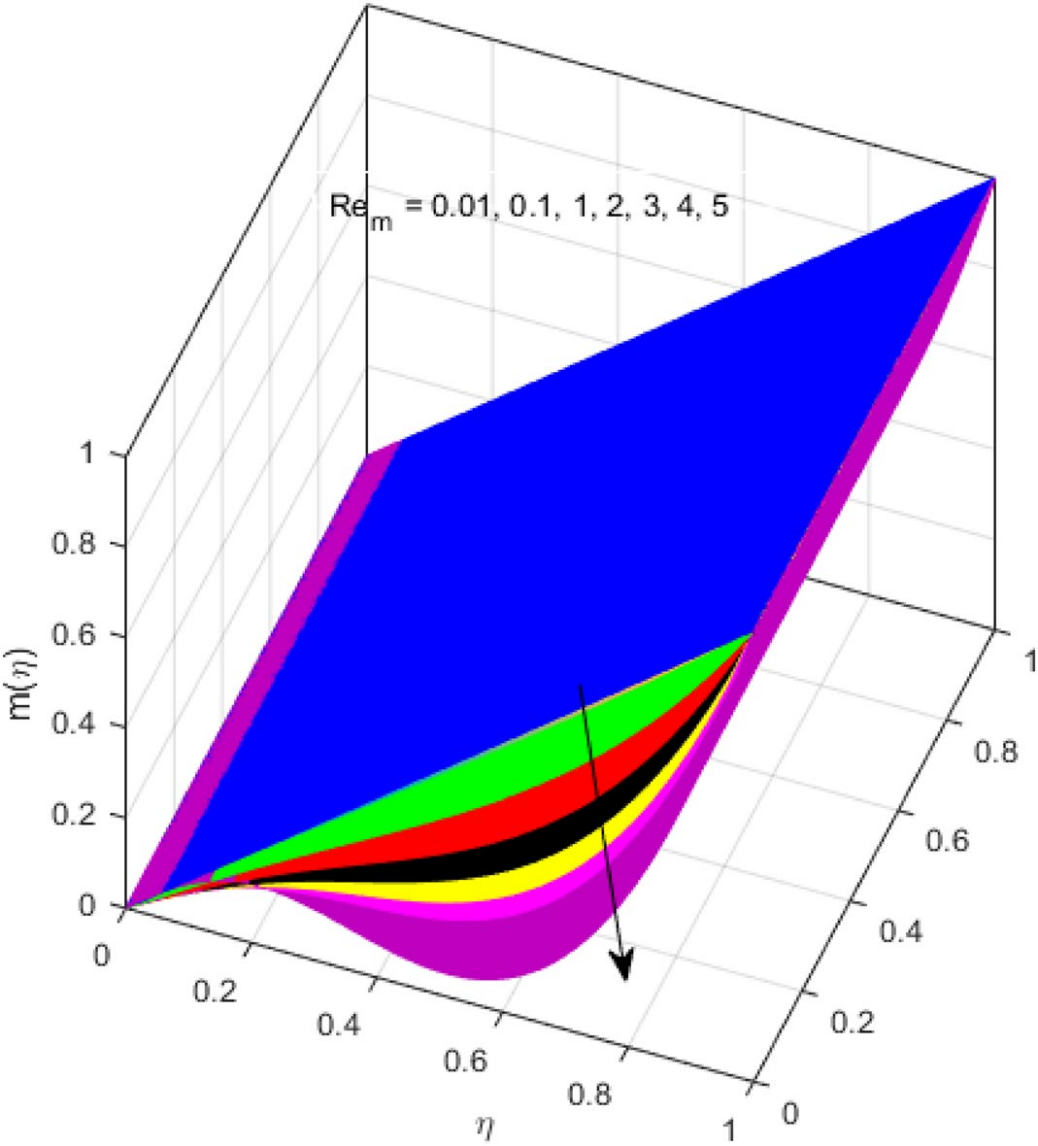
Axial component of an induced magnetic field for Re_*m*_. Image generated by using MATLAB 2015a https://www.mathworks.com/help/simulink/release-notes-R2015a.html.

**Figure 3:**
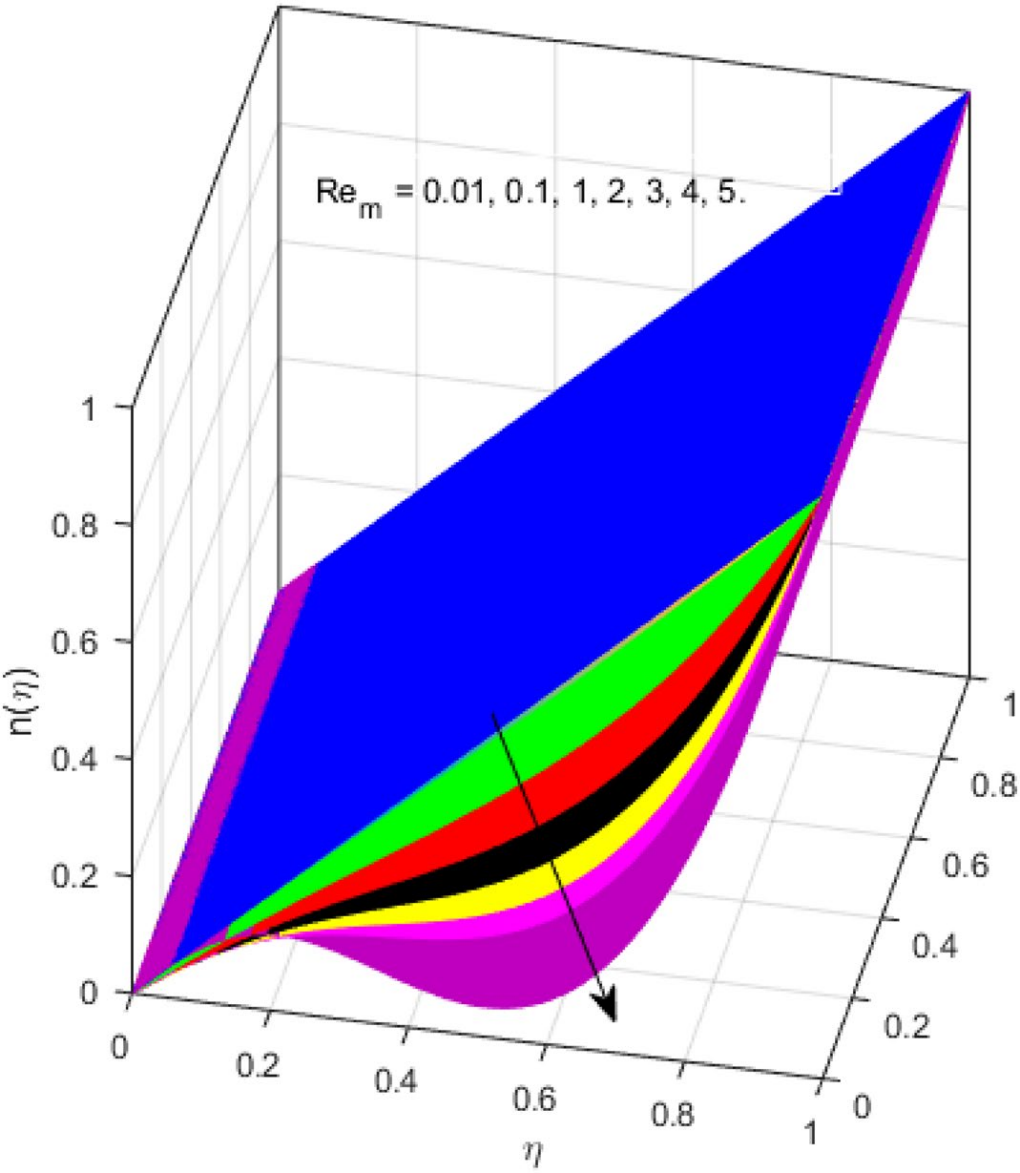
Tangential component of an induced magnetic field for Re_*m*_. Image generated by using MATLAB 2015a https://www.mathworks.com/help/simulink/release-notes-R2015a.html.

**Figure 4:**
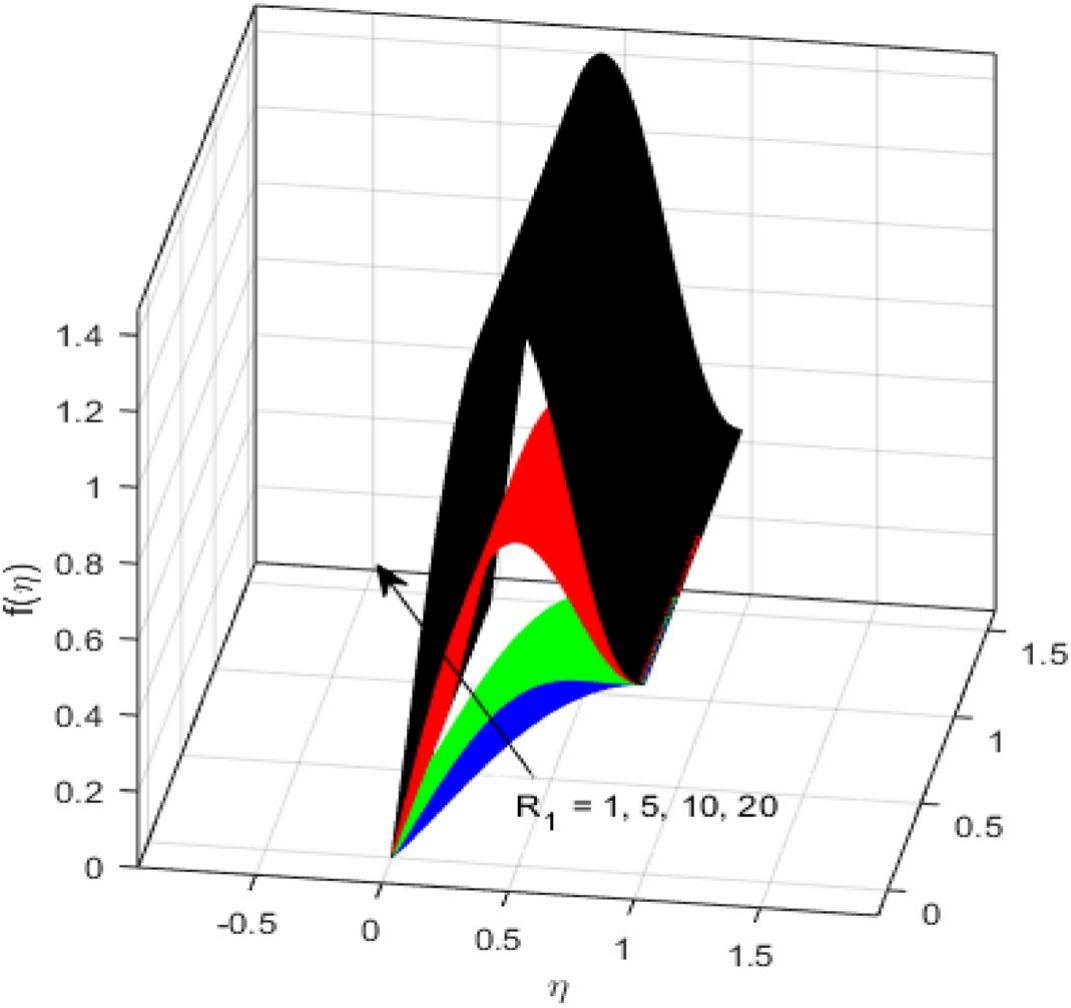
Axial velocity profile for *R*_1_. Image generated by using MATLAB 2015a https://www.mathworks.com/help/simulink/release-notes-R2015a.html.

**Figure 5:**
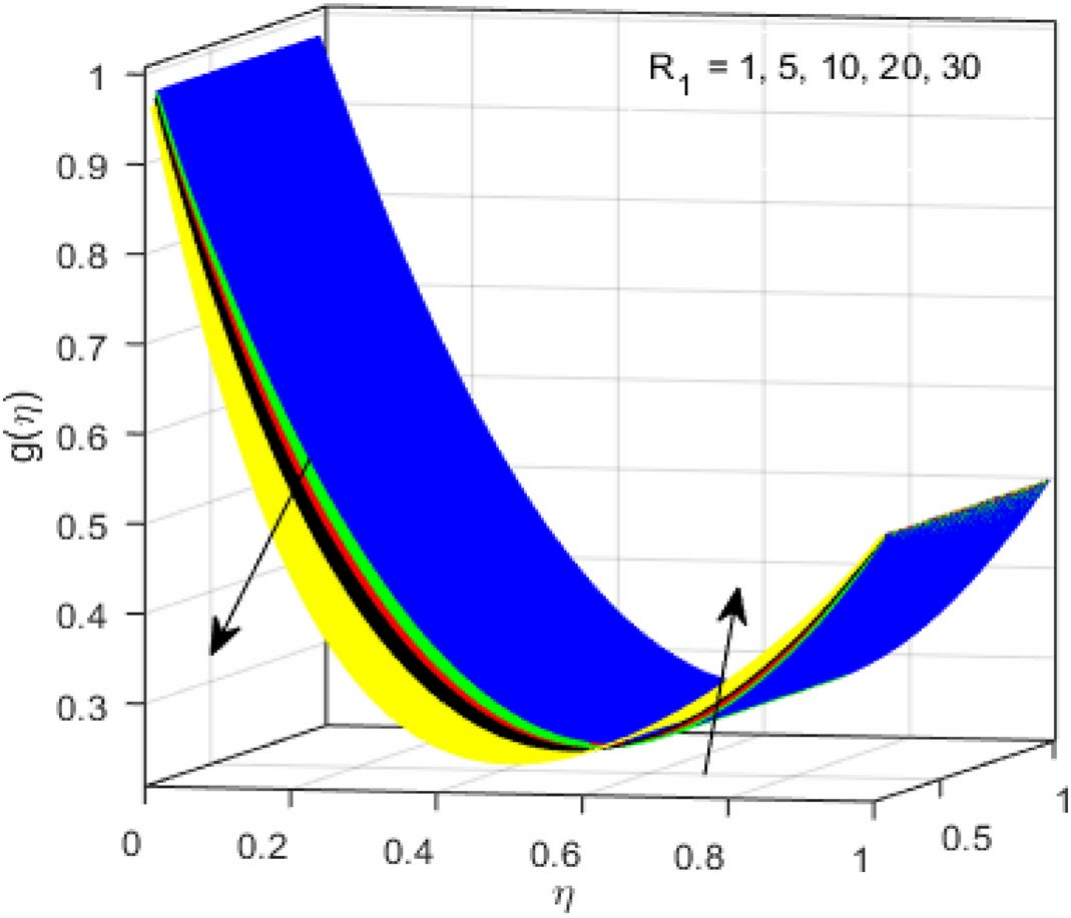
Tangential velocity profile for *R*_1_. Image generated by using MATLAB 2015a https://www.mathworks.com/help/simulink/release-notes-R2015a.html.

**Figure 6:**
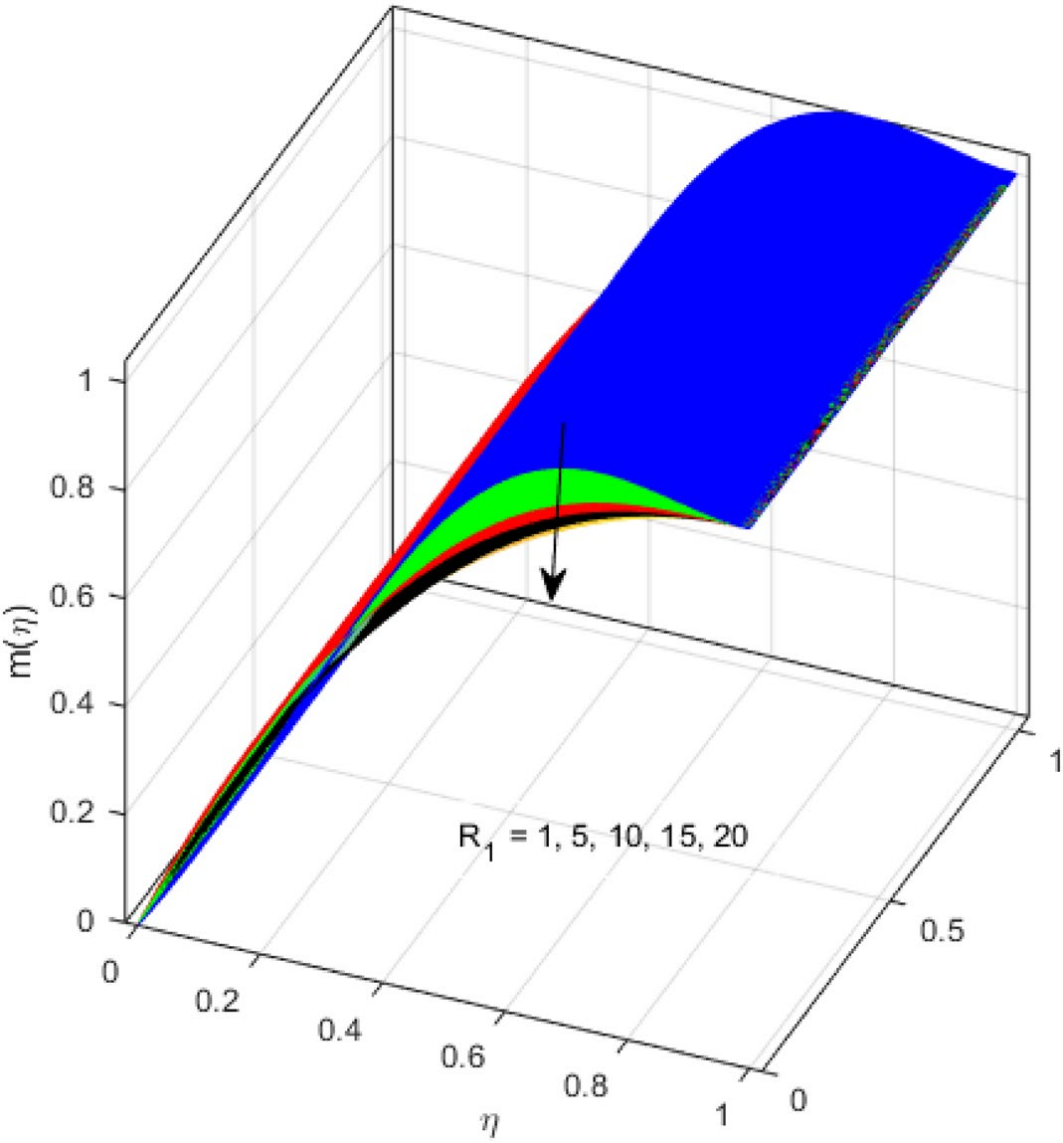
Axial component of induced magnetic field for *R*_1_. Image generated by using MATLAB 2015a https://www.mathworks.com/help/simulink/release-notes-R2015a.html.

**Figure 7:**
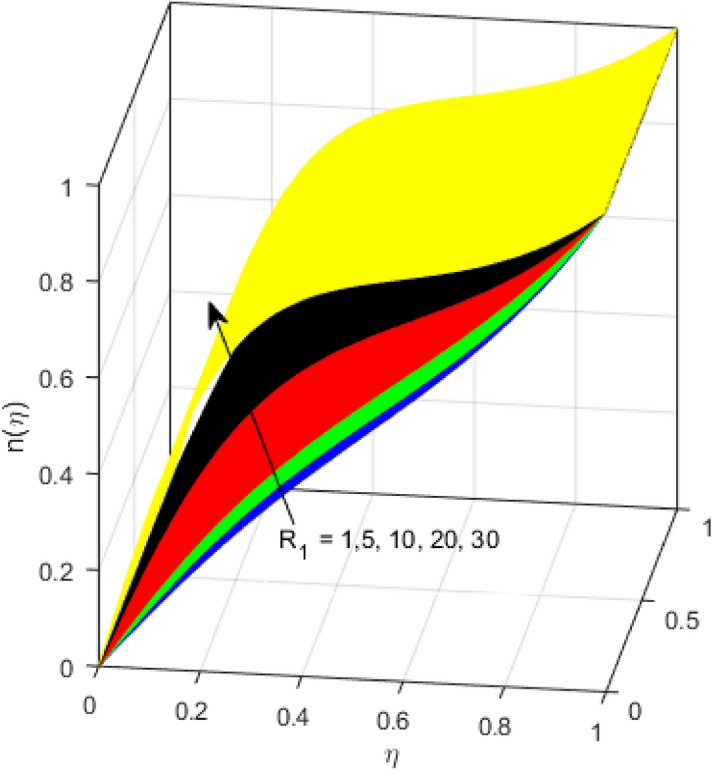
Tangential component induced magnetic field for *R*_1_. Image generated by using MATLAB 2015a https://www.mathworks.com/help/simulink/release-notes-R2015a.html.

**Figure 8:**
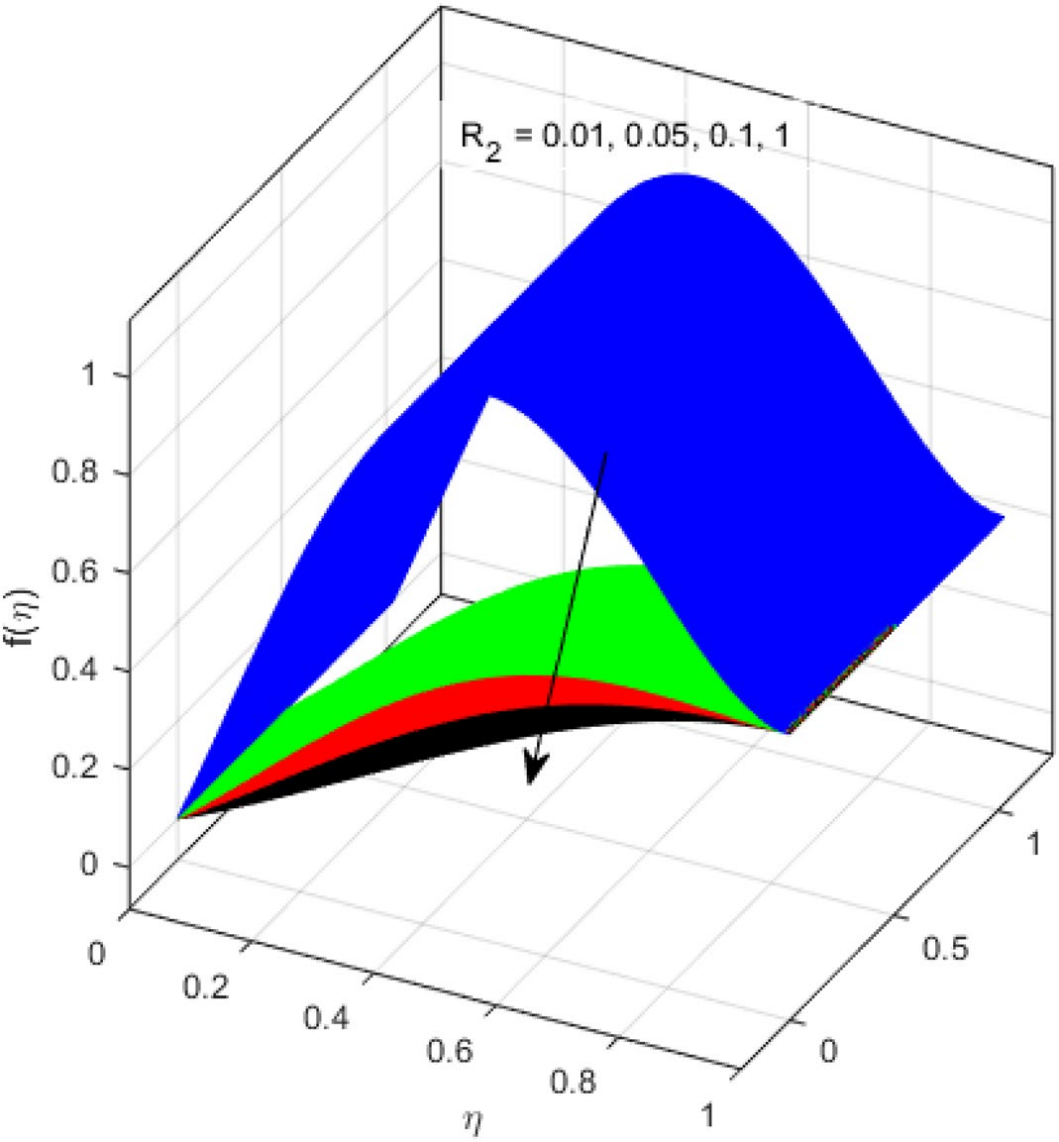
Axial velocity profile for *R*_2_. Image generated by using MATLAB 2015a https://www.mathworks.com/help/simulink/release-notes-R2015a.html.

**Figure 9:**
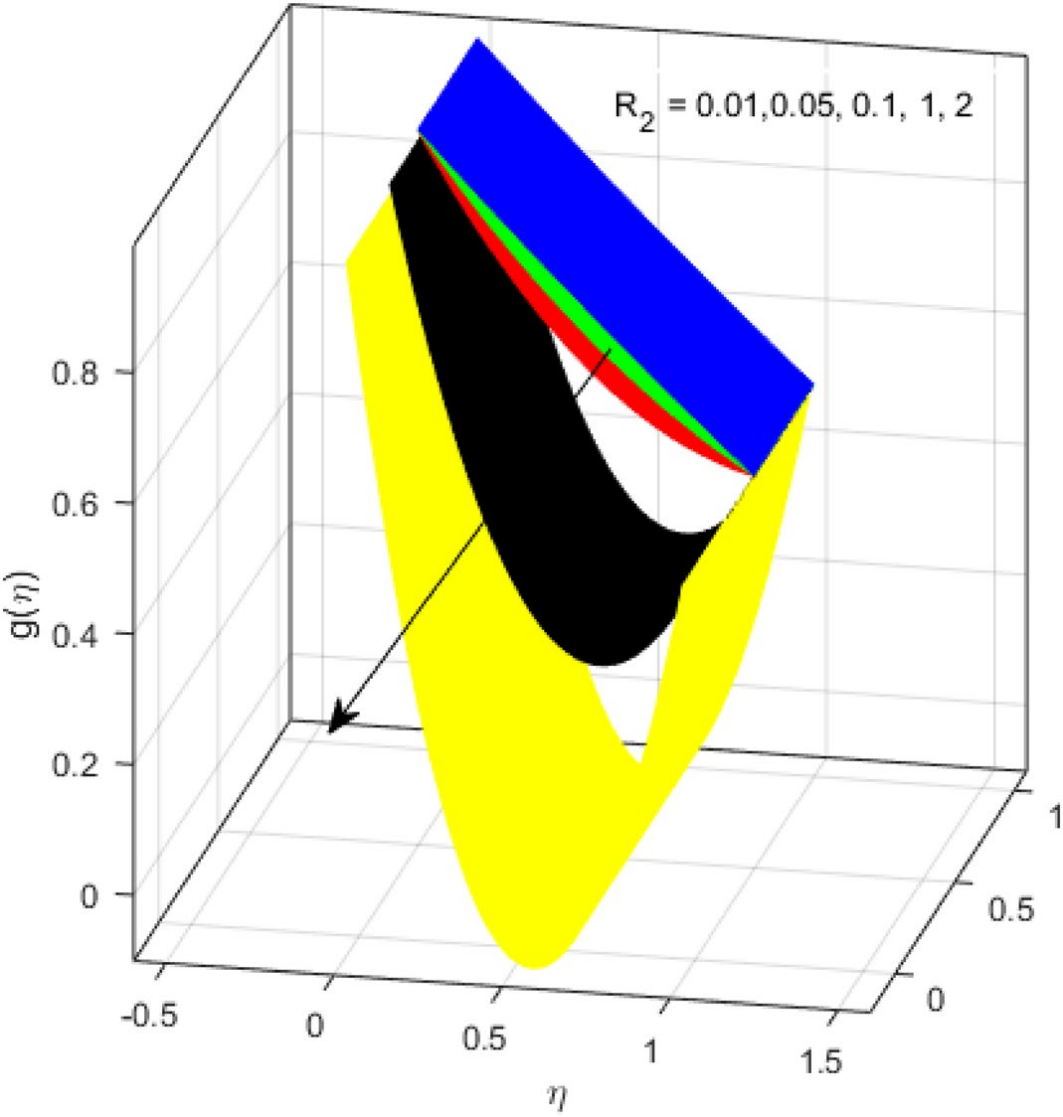
Tangential velocity profile for *R*_2_. Image generated by using MATLAB 2015a https://www.mathworks.com/help/simulink/release-notes-R2015a.html.

**Figure 10:**
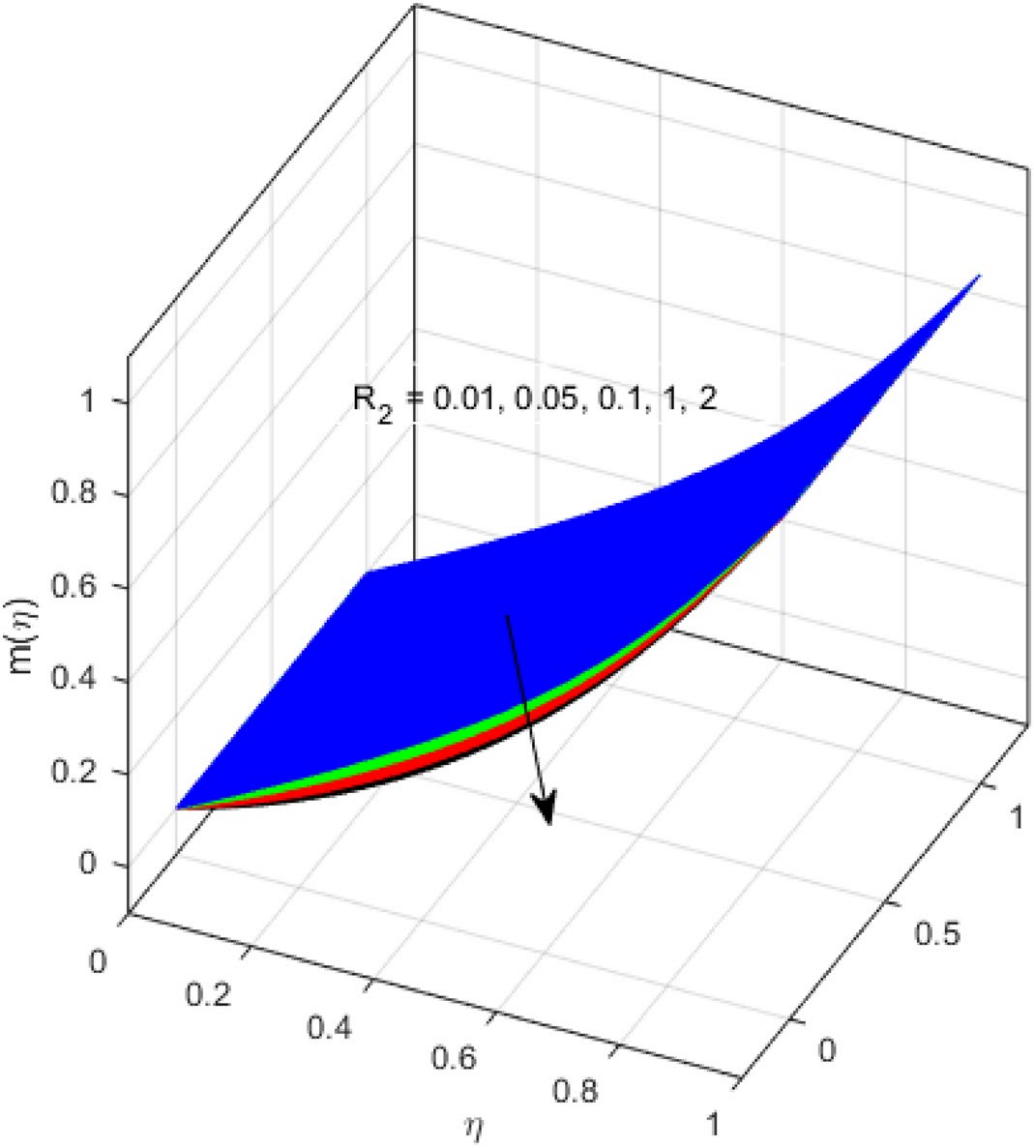
Axial component of induced magnetic field for *R*_2_. Image generated by using MATLAB 2015a https://www.mathworks.com/help/simulink/release-notes-R2015a.html.

**Figure 11:**
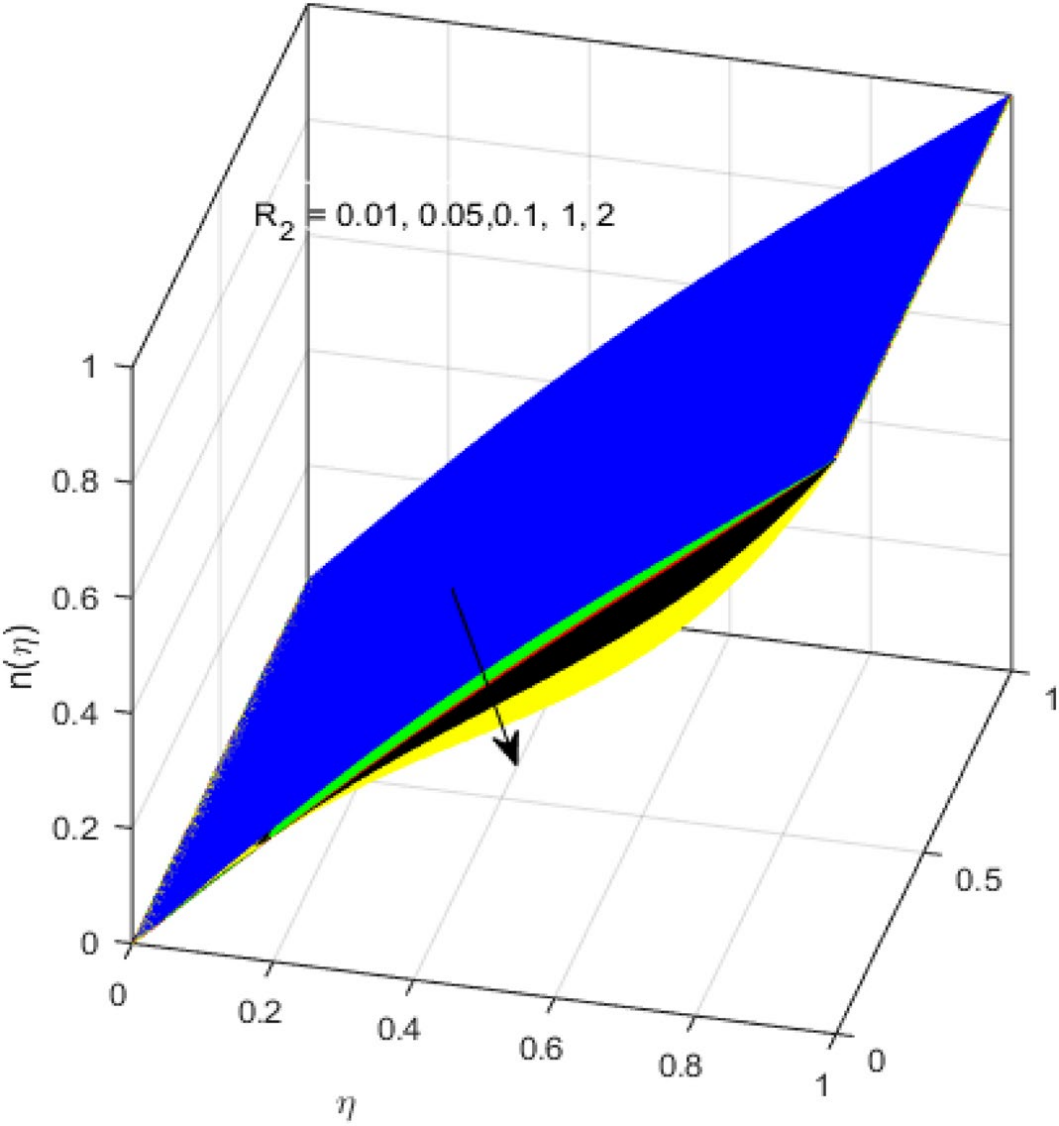
Tangential component of induced magnetic field for *R*_2_. Image generated by using MATLAB 2015a https://www.mathworks.com/help/simulink/release-notes-R2015a.html.

**Figure 12:**
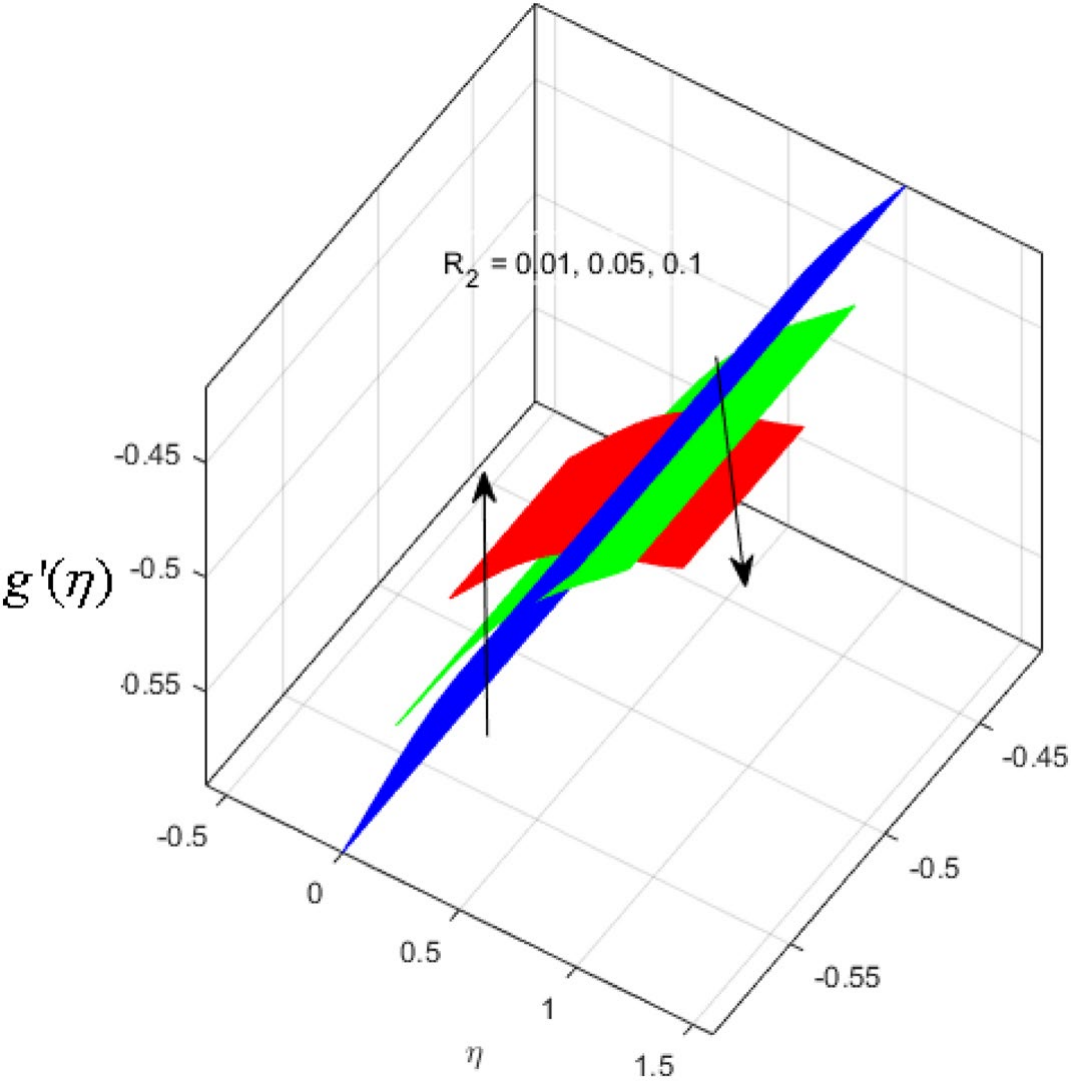
Upper disk torque for *R*_2_. Image generated by using MATLAB 2015a https://www.mathworks.com/help/simulink/release-notes-R2015a.html.

**Figure 13:**
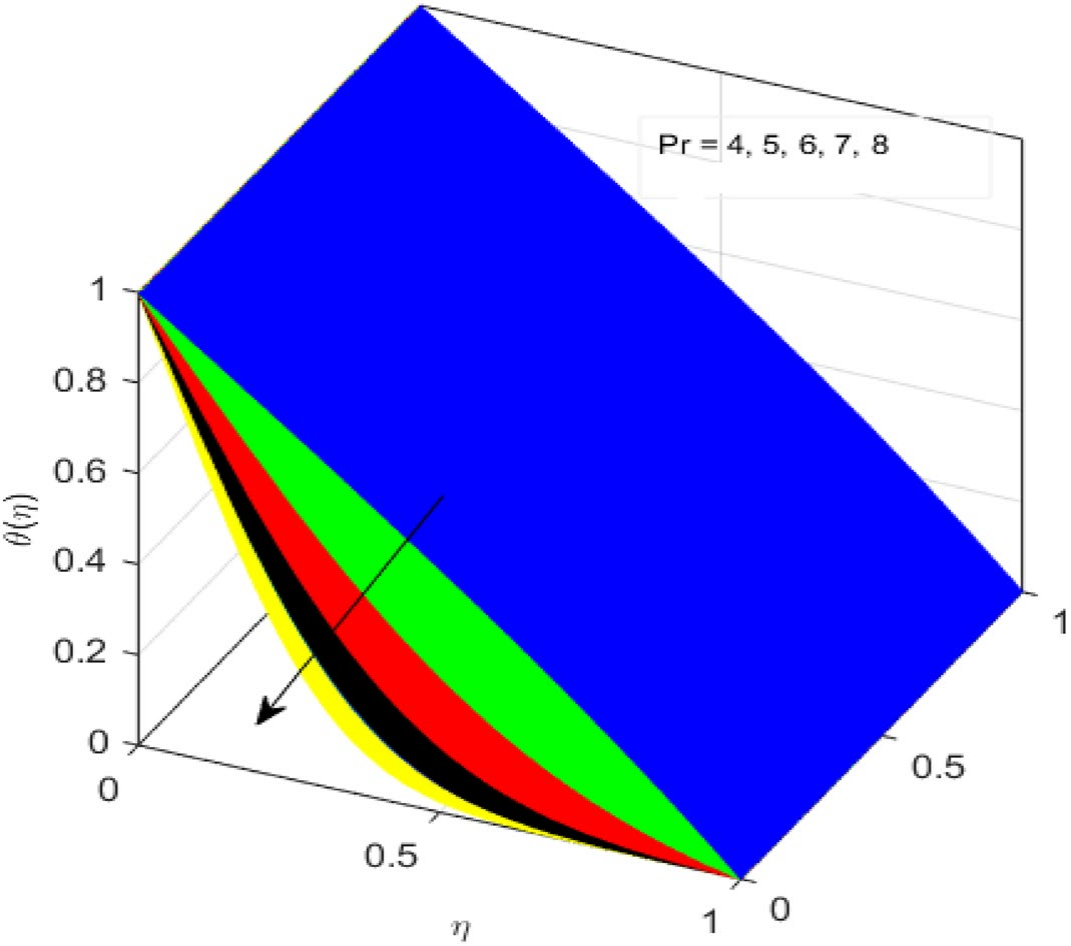
Variation of temperature profile for Pr. Image generated by using MATLAB 2015a https://www.mathworks.com/help/simulink/release-notes-R2015a.html.

**Figure 14:**
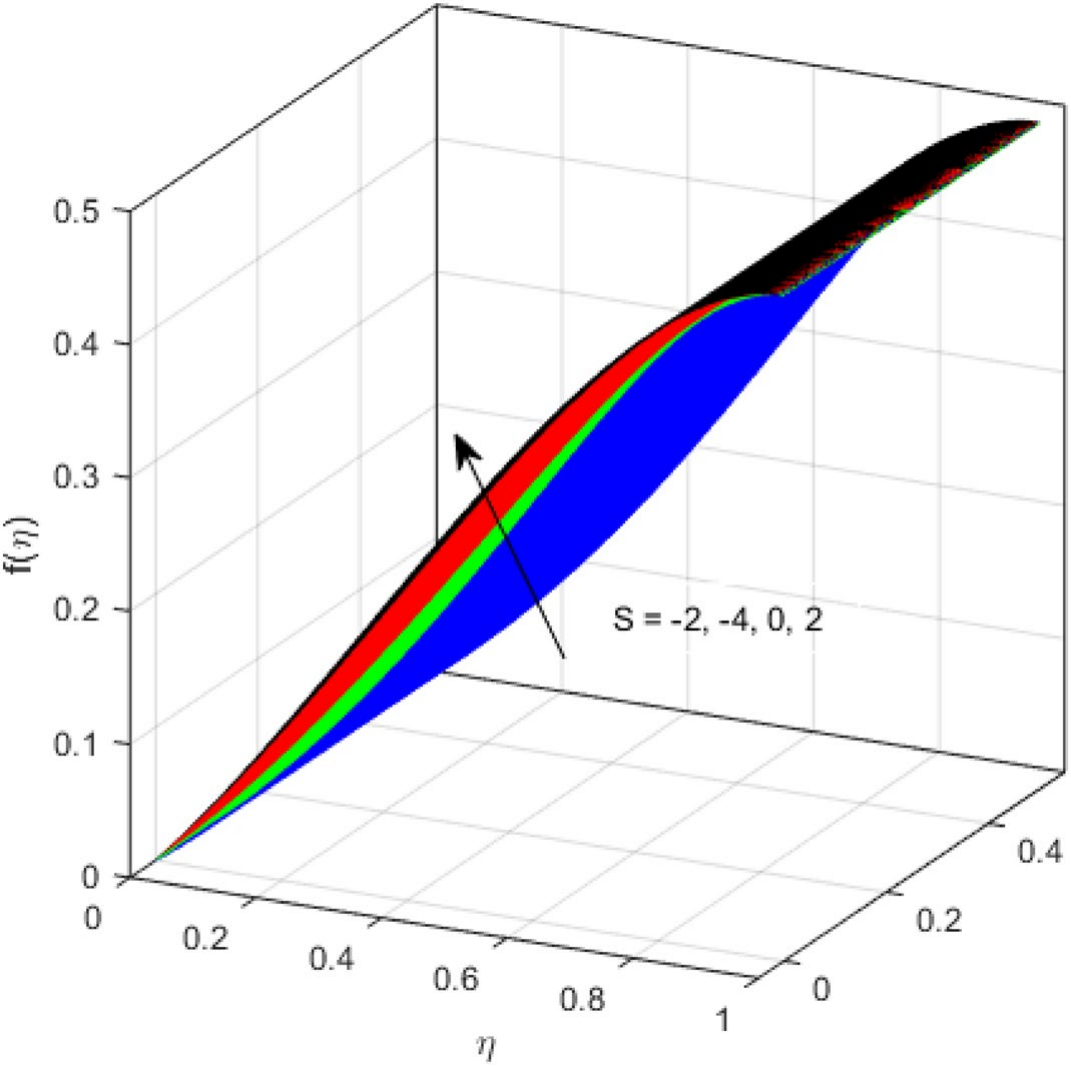
Axial velocity profile for *S*. Image generated by using MATLAB 2015a https://www.mathworks.com/help/simulink/release-notes-R2015a.html.

**Figure 15:**
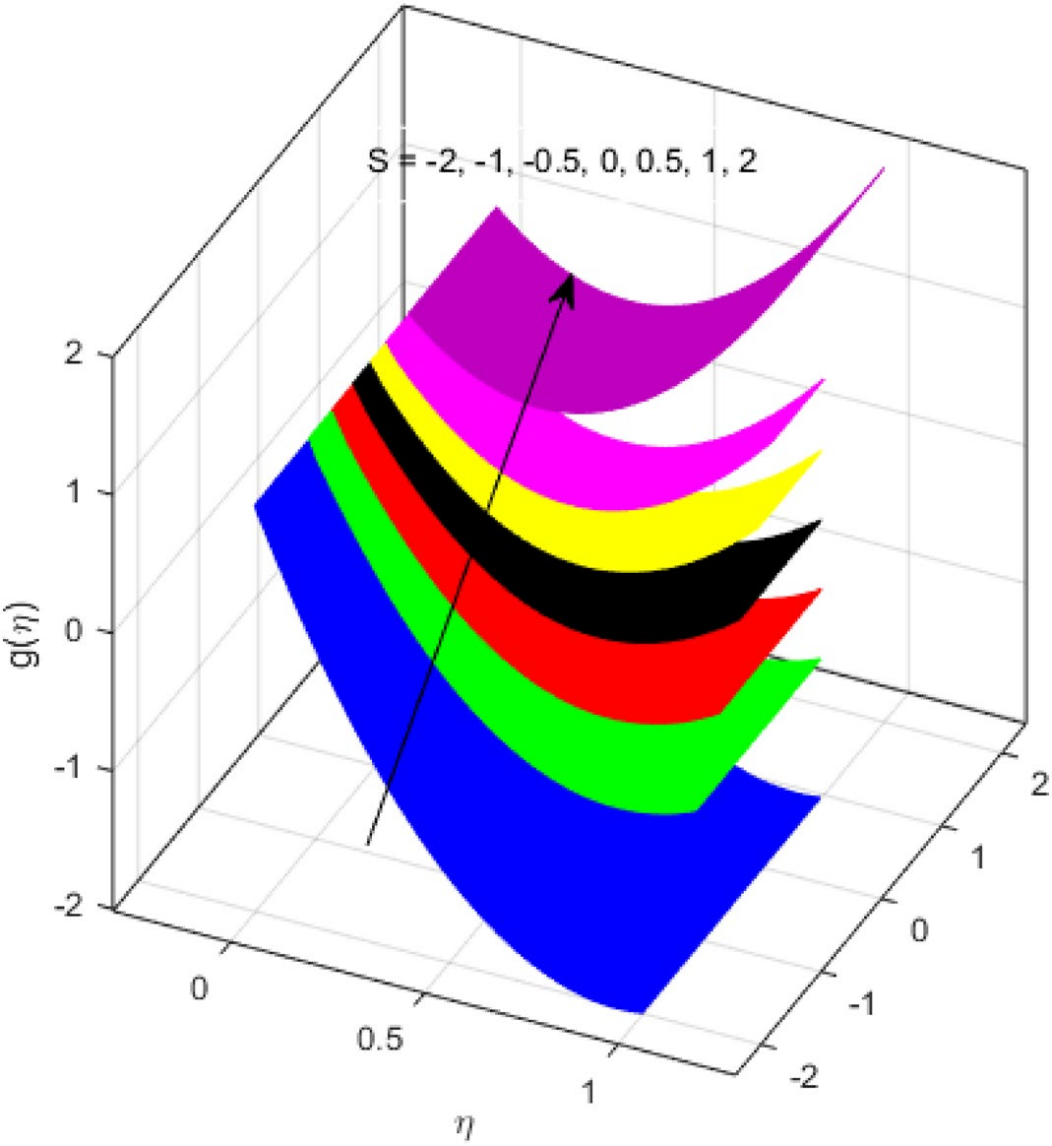
Tangential velocity profile for *S*. Image generated by using MATLAB 2015a https://www.mathworks.com/help/simulink/release-notes-R2015a.html.

**Figure 16:**
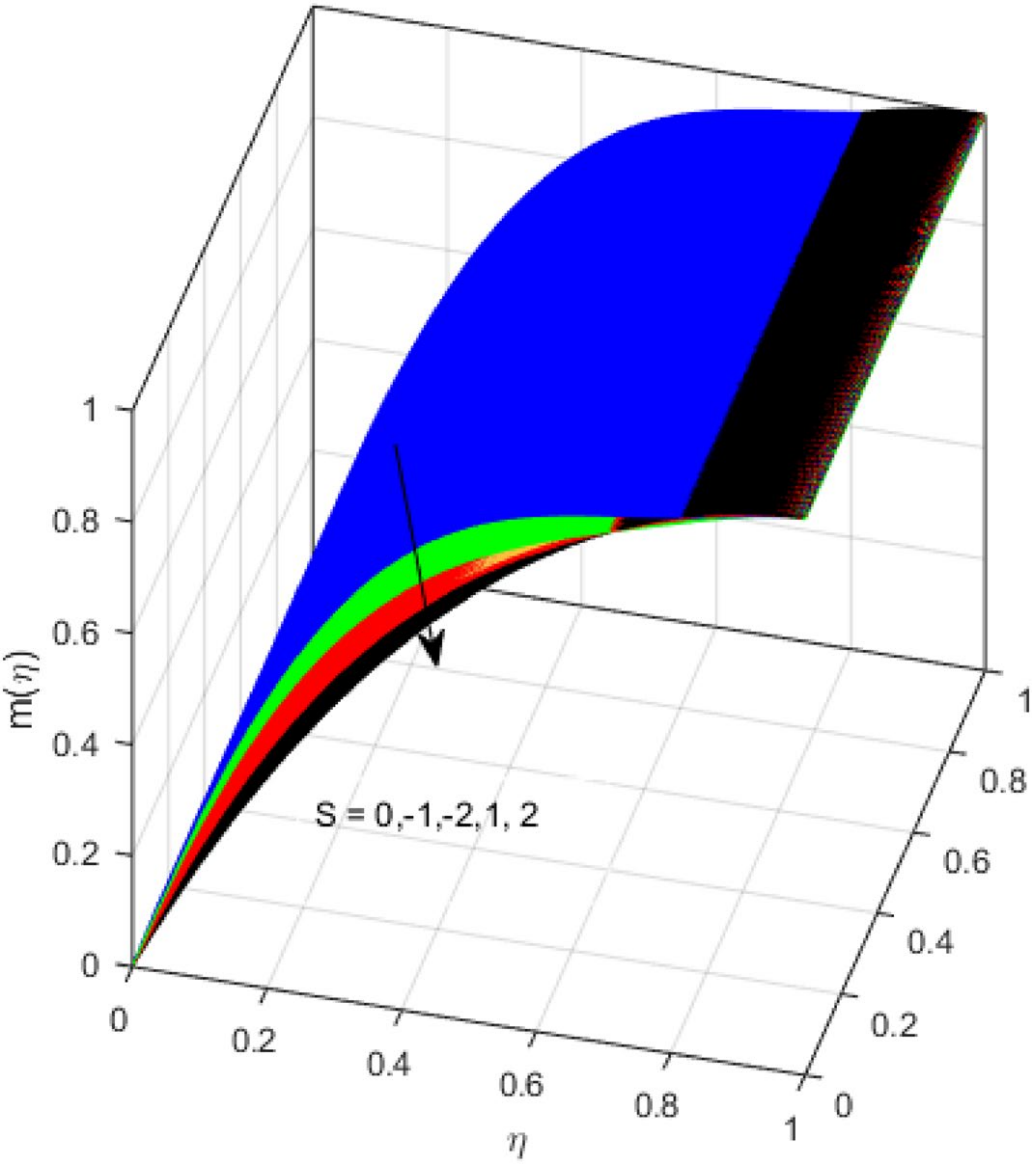
Axial induced magnetic field for *S*. Image generated by using MATLAB 2015a https://www.mathworks.com/help/simulink/release-notes-R2015a.html.

**Figure 17:**
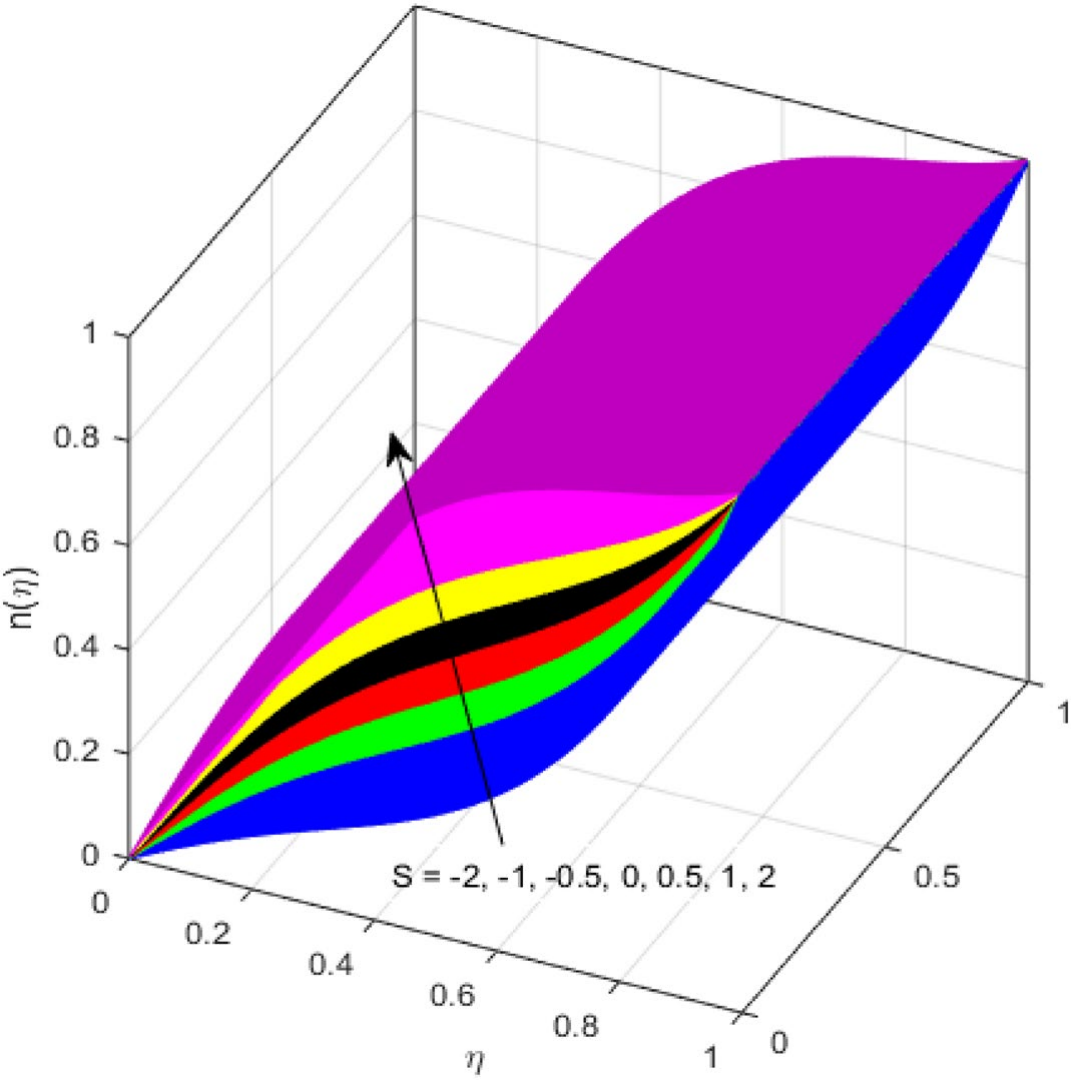
Tangential induced magnetic field for *S*. Image generated by using MATLAB 2015a https://www.mathworks.com/help/simulink/release-notes-R2015a.html.

**Figure 18:**
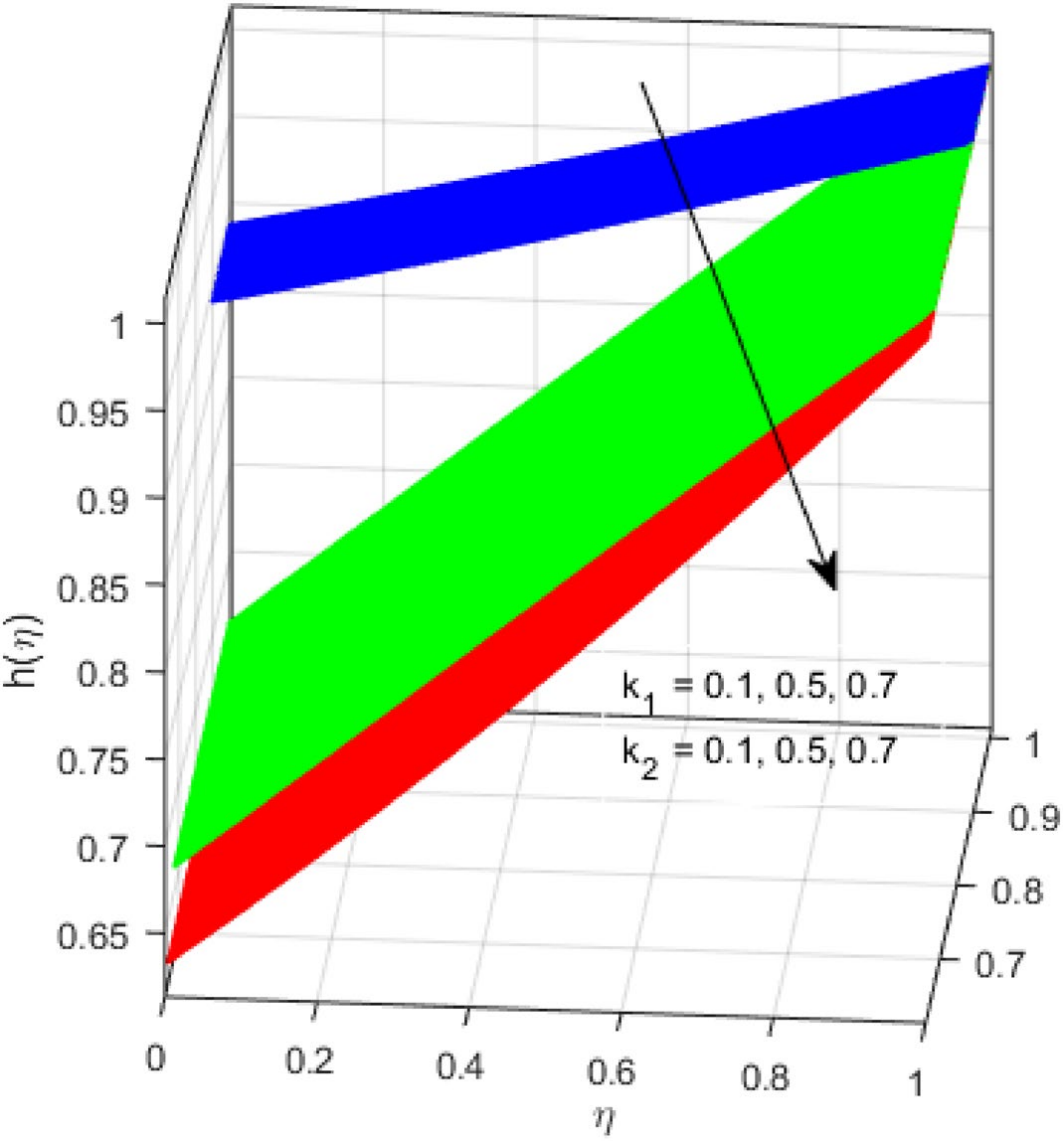
Variation of concentration profile for *k*_1_ and *k*_2_. Image generated by using MATLAB 2015a https://www.mathworks.com/help/simulink/release-notes-R2015a.html.

**Figure 19:**
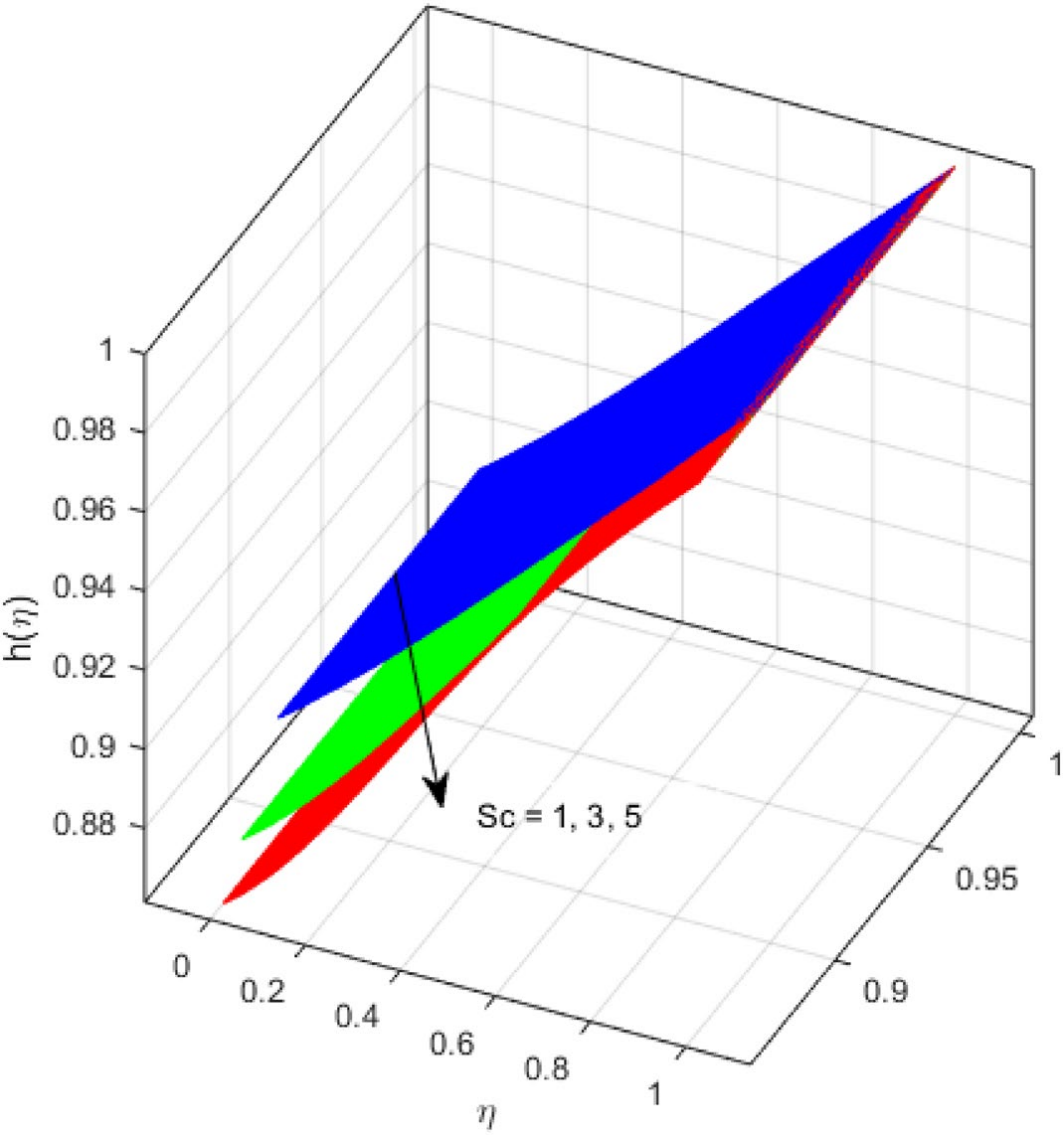
Variation of concentration profile for *Sc*. Image generated by using MATLAB 2015a https://www.mathworks.com/help/simulink/release-notes-R2015a.html.

**Figure 20:**
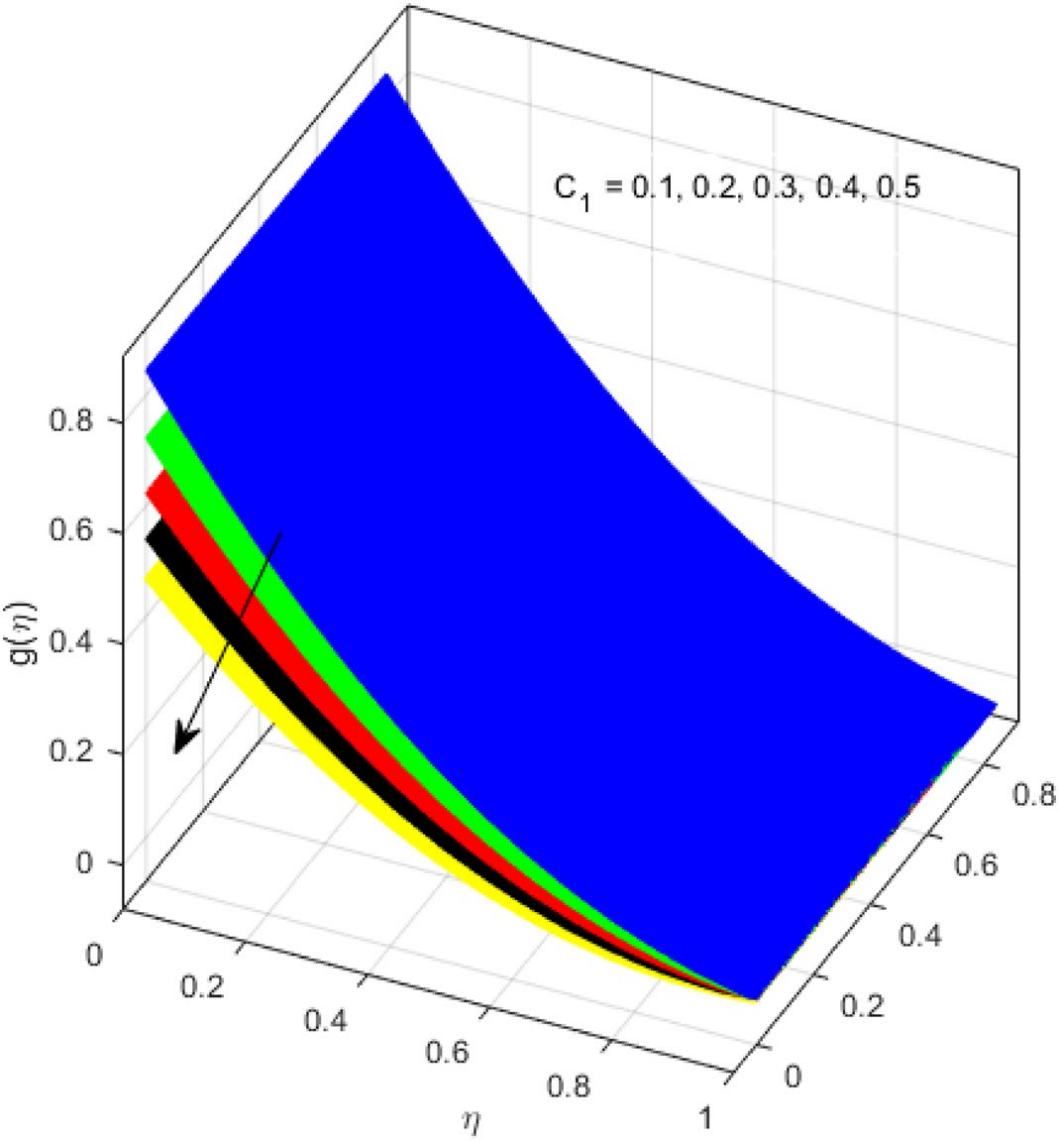
Variation of a tangential velocity profile for *C*_1_. Image generated by using MATLAB 2015a https://www.mathworks.com/help/simulink/release-notes-R2015a.html1.

This segment is devoted to the discussion on the impacts of numerous arising parameters on the associated profiles. The numerical values of arising pertinent parameters are kept fixed as $\lambda = 0.5,\,Fr = 0.5,\,k_{1} = 0.1,\,k_{2} = 0.1,\,R_{1} = 1,\,R_{2} = 0.01,\,R_{3} = 1,\,R_{4} = 0.5,\,{\text{Re}}_{m} = 0.1,\,Bt = 1,\,\Pr = 4,\,Sc = 1,\,S = 0.5$ otherwise stated. Figures 2 and 3 portray the impact of magnetic Reynolds number Re_*m*_ on the axial induced magnetic field *m*(*η*) and tangential (azimuthal) induced magnetic field *n*(*η*) respectively. It is witnessed that both *m*(*η*) and *n*(*η*) are decreasing functions for varied values of Re_*m*_. As the magnetic Reynolds number is the ratio of fluid flux to the mass diffusivity. Thus, by increasing Re_*m*_, a decrease in mass diffusivity and increase in fluid flux is seen. This decline in mass diffusivity disrupts the diffusion of the magnetic field and consequently, a decline in axial and tangential induced magnetic fields is witnessed. It is also observed that for small values of Re_*m*_ = 0.01, 0.1, both *m*(*η*) and *n*(*η*) are almost linear. However, for the values of Re_*m*_ = 1, 2, 3, 4, 5, there is a distortion in the induced magnetic field and thus the flow is greatly affected. The impact of the of rotational Reynolds number *R*_1_ on the axial velocity profile is depicted in Fig. 4. It is examined that the axial velocity profile ascends for higher rotational Reynolds number. The logic associated with this fact is that the axial velocity is generated owing to the vertical movement of the upper disk and the radial flux near the lower disk far from the axis of rotation. It further observed from Fig. 4 that there is a gradual increase in the axial velocity profile for *R*_1_ = 1–10, and a sharp increase in the amplitude in axial velocity curve for *R*_1_ = 20, when *η* = 0–5. This parabolic trend at *R*_1_ = 20 is called Critical value of rotational Reynolds number. Figure 5 exhibits the response of tangential velocity profile *g*(*η*) for varying *R*_1_. It is seen that the tangential velocity profile has a maximum amplitude in the vicinity of the lower disk at *η* = 0 by fixing the `rotation parameter *S* = 0.5. Since, Ω_2_ = 0.5Ω_1_, (where Ω_2_, and Ω_1_ are angular velocities of the lower and upper disks respectively), which means the angular velocity of the lower disk is half of the upper disk. Thus, increasing the rotational Reynolds number, Ω_1_ significantly increases. So closer to the lower disk, tangential velocity decrease for the growing values of *R*_1_ and hence maximum amplitude is observed near the lower disk. The behavior of the axial induced magnetic field *m*(*η*) for growing values of rotational Reynold number *R*_1_ is exhibited in Fig. 6. It is witnessed that the *m*(*η*) rises in the vicinity of the lower disk and descends near the upper disk. The increasing values of *R*_1_ causes an increase in the angular velocity of the upper disk which affects the magnetic field along the streamlines. Thus, significant curved behavior is witnessed near the upper disk in comparison to the lower one. Figure 7 is illustrated for varied estimates of rotational Reynold number *R*_1_ versus tangential induced magnetic field *n*(*η*). It is noted that *n*(*η*) increases throughout the system for varying values of *R*_1_. Higher estimates of *R*_1_ causes a rise in the radial flux far from the axis of rotation which ultimately boosts the tangential induced magnetic field. The impact of the axial velocity profile *f*(*η*) for increasing squeezed Reynolds number *R*_2_ is depicted in Fig. 8. It is understood from the graph behavior that *f*(*η*) decreases with increasing estimates of *R*_2_. The large estimates of squeezed Reynolds number *R*_2_ boosts the magnetic Reynolds number Re_*m*_ that ultimately affects the mass diffusivity because magnetic Reynolds number is the ratio of the fluid flux to mass diffusivity. That is why decreasing the behavior of the axial velocity profile is witnessed. Figure 9 is plotted to observe the trend of the tangential velocity profile *g*(*η*) for increasing values of squeezed Reynolds number *R*_2_. From the graphical illustration, it is understood that *g*(*η*) is declining function of *R*_2_. The graph shows a significant change from linear to curved once the values of *R*_2_ are swapped from the smaller to the larger. Large estimates of *R*_2_ disrupt the mass diffusivity that eventually lowers the tangential velocity profile. The outcomes of the squeezed Reynolds number *R*_2_ on the induced magnetic field *m*(*η*) and tangential induced magnetic field *n*(*η*) are depicted in Figs. 10 and 11 respectively. It is seen that both *m*(*η*) and *n*(*η*) show declined trend versus large estimates of *R*_2_. The increasing squeezed Reynolds number is related to the upsurge in the magnetic Reynolds number. As the fluid flow in a magnetic field is characterized by the value of the magnetic Reynolds number. So, by increasing *R*_2_, fluid flux to mass diffusivity ratio increases which results in a decrease in both profiles *m*(*η*) and *n*(*η*) Fig. 12 depicts the variation in torque *g*′(*η*) for increasing positive values of squeeze Reynolds number *R*_2_. The growing estimates of the squeezing Reynolds number strengthen the magnetic force in both axial and tangential directions. As the angular velocity of the upper disk is twice the velocity of the lower disk. So, the torque decreases in the vicinity of the upper disk, and an opposite trend is witnessed in the vicinity of the lower disk. Figure 13 is drawn to see the variation in temperature profile for varied values of the Prandtl number. It is comprehended that the Prandtl number causes a decline in the thermal profile. As the Prandtl number is the ratio of momentum to thermal diffusivity. The large estimates of the Prandtl number mean the weaker thermal diffusivity. Hence heat diffuses gradually and thus affecting the temperature. The influence of the disks’ rotational velocity ratio parameter *S* on the axial *f*(*η*) and the tangential *g*(*η*) velocity profiles is given in Figs. 14 and 15 respectively. An increase in both velocity distributions is observed for numerous estimates of *S*. It is pertinent to mention that *S* = 1, and *S* =  − 1, correspond to the clockwise and counterclockwise rotation of the disks for the same angular velocity. And *S* = 0, relates to the no rotation case. Figures 16 and 17 exhibit the variation of the axial induced magnetic field *m*(*η*) and tangential induced magnetic field *n*(*η*) for the rotational velocity ratio parameter *S*, respectively. Here, all three cases *i.e.,* clockwise, anticlockwise, and no rotation, are discussed. It is witnessed that the axial induced magnetic field declines for all the cases (Fig. 16). It is also observed that the maximum value of the axial component of the induced magnetic field occurs for the non-rotation case. The tangential induced magnetic field also increases for all the cases (Fig. 17). Increasing values of *S* means the angular velocity of the upper disk is less than the lower disk, which causes the increase in the tangential component of the magnetic force. The impact of the homogeneous and heterogeneous reaction parameters on the concentration of the nanofluid thin-film flow is portrayed in Fig. 18. It is comprehended that the concentration profile declines for growing estimates of both parameters. As the reaction proceeds, the reactants are consumed which causes the concentration profile to decrease. Figure 19 indicates the variation in the concentration profile for increasing Schmidt number. As Schmidt number is the quotient of momentum to mass diffusivity. Thus, smaller mass diffusivity relates to stronger Schmidt number, so the decrement of mass diffusivity results in the decrease in the concentration profile. To analyze the impact of velocity slip parameter on tangential velocity profile Fig. 20 is drawn. It is visualized that tangential velocity decrease for mounting values of the velocity slip parameter. In fact, with an increase in the velocity slip parameter, stretching velocity is partially transferred to the fluid. So, velocity declines. The majority of the above outcomes apart from the new suppositions exactly correlate to the results obtained by Zueco et al.^26^.

Table 2 depicts the numerical values of the torque *g*′(*η*) by fixing *R*_2_ = 1, *R*_4_ = 0.5, *Bt* = 0.6, *A*_1_ = 0, and *C*_1_ = 0, varying *R*_3_. The results are compared with Rashidi et al.^27^. An excellent agreement between the values is attained. It is comprehended from Table 2 that by taking *R*_3_ > *R*_4_ and by varying *R*_2_ increase in the torque of both disks is witnessed. However, the torque of the lower disk is higher than the upper one. Table 3 portrays the numerical values of the torques $\frac{dg(0)}{{d\eta }}$ and $\frac{dg(1)}{{d\eta }}$ by fixing *R*_2_ = 1, *R*_4_ = 0.5, *Bt* = 0.6, *A*_1_ = 0, and *C*_1_ = 0, varying *R*_2_. The results are compared with Rashidi et al.^27^. An excellent agreement between the values is attained. Table 3 depicts that if we consider *R*_2_ < *R*_4_ < *R*_3_ and vary the values of *R*_2_, then the torque at lower disk increases while the torque at the upper disk diminishes.

**Table 2:** Comparison of torques $\frac{dg(0)}{{d\eta }}$ and $\frac{dg(1)}{{d\eta }}$ at the lower and upper disks for varied values of *R*_3_ with Rashidi et al.^27^, when *R*_2_ = 1, *R*_4_ = 0.5, *Bt* = 0.6, *A*_1_ = 0, and *C*_1_ = 0.

*R*_3_	$\frac{dg(0)}{{d\eta }}$			$\frac{dg(1)}{{d\eta }}$		
	DTM-Pade	Numerical result	Our results @@Bvp4c	DTM-Pade	Numerical result	Our results@@Bvp4c
0	− 1.77239023	− 1.77238909	− 1.77238908	− 0.60923829	− 0.60923369	− 0.60923368
1	− 1.79906809	− 1.79906821	− 1.79906820	− 0.89280560	− 0.89280536	− 0.89280531

**Table 3:** Comparison of torque $\frac{dg(0)}{{d\eta }}$ and $\frac{dg(1)}{{d\eta }}$ at the lower and upper disks for varied values of *R*_2_ with Rashidi et al.^27^, when *R*_3_ = 1, *R*_4_ = 0.5, *Bt* = 0.6, *A*_1_ = 0, and *C*_1_ = 0.

*R*_2_	$\frac{dg(0)}{{d\eta }}$			$\frac{dg(1)}{{d\eta }}$		
	DTM-Pade	Numerical result	Bvp4c method @@Our results	DTM-Pade	Numerical result	Bvp4c method@@Our results
0.1	− 1.08963495	− 1.08963506	− 1.08963501	− 0.95987351	− 0.95987349	− 0.95987348
0.2	− 1.17203735	− 1.17203765	− 1.17203761	− 0.93844853	− 0.93844830	− 0.93844829

## Final comments

In this exploration, we have studied the squeezing three-dimensional hydromagnetic nanofluid thin-film flow amid two rotating disks in a Darcy–Forchheimer permeable media. The purpose of using nanofluid with multiwalled carbon nanotubes is to get better thermal conductivity in the presence of an induced magnetic field. The analysis is done by invoking partial slip effect at the boundary in the presence of autocatalytic chemical reactions. The mathematical model consists of axial and tangential momentum and magnetic fields respectively. The tangential and axial velocity distributions and components of the magnetic field are examined numerically by employing the bvp4c method for varying magnetic, rotational, and squeezing Reynolds number. The flow field is governed by the squeezed, rotational, and magnetic Reynolds number. The main findings of our observations in light of the above-raised questions are appended as below:

- The increasing magnetic Reynolds number reduces the axial and azimuthal components of the induced magnetic field.
- An increase in the axial velocity profile is seen for increasing the rotation parameter.
- For the higher value of magnetic Reynolds number, the squeezed film possesses the much higher thermal conductivity in the presence of the induced magnetic field. This significant increase in the thermal conductivity is because of the nanofluid with suspended multiwalled carbon nanotubes.
- Increasing the Prandtl number causes the thermal profile to decrease.
- The tangential velocity profile boosts for varied estimates of relative rotation parameter.
- The concentration of the nanofluid flow is a declining function of the autocatalytic chemical reactions.

## List of symbols

*A**: Chemical specie
*a*: Concentration of chemical specie *A**
*B**: Chemical specie
*b*: Concentration of chemical specie *B**
Bt: Batcheler number
***B*** = (*B*_*r*_, *B*_*θ*_, *B*_*z*_): Induced magnetic field
*D*: Length equivalent to disk separation at *t* = 0
*f*: Axial velocity
*F*_*r*_: Forchheimer number
***H*** = (*H*_*θ*_, *H*_*z*_): Externally applied magnetic field
*k*_1_, *k*_2_: The measure of the strength of homogeneous and heterogeneous reactions respectively
*m*: Axial induced magnetic field component
*n*: Azimuthal induced magnetic field component
*M*_*o*_, *N*_*o*_: Magnetic field quantities
Pr: Prandtl number
*r*: Radial coordinate
*R*_1_: Rotational Reynolds number
*R*_2_: Squeezed Reynolds number
*R*_3_: Dimensionless parameters for magnetic force in an axial direction
*R*_4_: Dimensionless parameters for magnetic force in a tangential direction
Re_*m*_: Magnetic Reynolds number
$S = \frac{{\Omega_{2} }}{{\Omega_{1} }}$: Disks rotational velocity ratio
*Sc*: Schmidt number
***V*** = (*u*, *v*, *w*): Velocity vector in (*r*, *θ*, *z*) direction
*z*: Axial coordinate

## Greek letters

Ω_1_: Angular velocity of the lower disk
Ω_2_: Angular velocity of the upper disk
*μ*_2_, *μ*_1_: Magnetic permeabilities of squeezed film and medium external to the disk
*λ*: Porosity parameter
*μ*_nf_: Dynamic viscosity of nanofluid
*θ*: Tangential coordinate
∇: Vector field operator
*ζ*: Dimensionless time
*η*: Dimensionless z- coordinate
*σ*_*nf*_: Electrical conductivity of nanofluid
*α*: Inverse time, *t* = *α*^−1^
